# Adverse event mining for Breztri and Trelegy Ellipta based on the three international pharmacovigilance databases

**DOI:** 10.1097/MD.0000000000049162

**Published:** 2026-06-05

**Authors:** Junyu Wang, Kexu Chen, Lu Yun, Dexiang Xu

**Affiliations:** a Department of Pharmacy, Qingdao Central Hospital, University of Health and Rehabilitation Sciences, Qingdao, China; b Department of Nuclear Medicine, Qingdao Central Hospital, University of Health and Rehabilitation Sciences, Qingdao, China; c Department of Pulmonary and Critical Care Medicine, Qingdao Central Hospital, University of Health and Rehabilitation Sciences, Qingdao, China.

**Keywords:** adverse drug reaction study, breztri, disproportionality analysis, FAERS database, trelegy ellipta

## Abstract

This study aimed to identify adverse drug reaction (ADR) risk signals associated with budesonide/glycopyrrolate/formoterol (Breztri) and fluticasone furoate/umeclidinium/vilanterol (Trelegy Ellipta) to support clinical decision-making and risk management. ADR reports related to Breztri and Trelegy Ellipta from the FDA Adverse Event Reporting System (FAERS) database, Japanese Adverse Drug Event Report (JADER) database and Canada Vigilance Adverse Reaction database (Q3 2004 to Q2 2024) were analyzed. After deduplication, reports were categorized using Medical Dictionary for Regulatory Activities (MedDRA) to obtain System Organ Class (SOC) and preferred terms (PTs). Disproportionality analysis was conducted using reporting odds ratio (ROR) and proportional reporting ratio methods. Analysis of 394 Breztri and 18,866 Trelegy Ellipta FAERS reports (predominantly consumer-submitted, U.S.-originated) identified 47 signals across 11 SOCs for Breztri (e.g., “Drug delivery system issue” ROR = 411.16, “Intentional device misuse” ROR = 410.69) and 160 signals across 15 SOCs for Trelegy (e.g., “Chronic eosinophilic rhinosinusitis” ROR = 187.65, “Foreign body in mouth” ROR = 107.67), revealing unlabeled risks like administration errors and packaging confusion. JADER data reinforced respiratory risks (Breztri: Chronic obstructive pulmonary disease (COPD) ROR = 516.8; Trelegy: gastrointestinal fungal infection ROR = 413.11) and device-independent safety signals (e.g., Trelegy urinary retention ROR = 28.81), while CVARD highlighted region-specific concerns including Trelegy-associated vasculitis (pulmonary vasculitis n = 29) and Breztri hypertension (ROR = 7.82). Cross-database convergence confirmed core anticholinergic/cardiopulmonary risks, yet divergent signals, FAERS’ device errors, JADER’s infection prominence, and CVARD’s immunological events, underscore geographic heterogeneity in adverse reaction profiles, necessitating tailored risk management strategies for inhaler therapies. Inhalation device-related ADRs were observed, with Breztri showing higher incidence than Trelegy Ellipta, likely due to its more complex device usage. These findings highlight the need for enhanced patient education by healthcare providers to ensure proper device use in COPD treatment. Although core respiratory and anticholinergic risks are globally relevant, infection profiles, device complications, and rare immunological events exhibit significant geographic heterogeneity, necessitating tailored risk mitigation strategies aligned with regional pharmacovigilance patterns.

## Abstract 2

This study aimed to identify adverse drug reaction (ADR) risk signals associated with Breztri and Trelegy Ellipta to support clinical decision-making and risk management.

For Breztri, 394 ADR reports were identified, mostly submitted by consumers (224, 56.9%) and originating from the United States (386, 98%). Signal analysis identified 47 positive signals across 11 SOCs, with common ADRs including “Drug delivery system issue” and “Intentional device misuse.” For Trelegy Ellipta, 18,866 ADR reports were retrieved, predominantly from consumers (16,413, 87%) in the United States (16,004, 84.8%). Signal analysis identified 160 positive signals across 15 SOCs, including “Chronic eosinophilic rhinosinusitis” and “Foreign body in mouth.” Both drugs revealed new potential risks, such as incorrect administration duration, packaging name confusion, and dosage errors, not listed in drug labels.

Inhalation device-related ADRs were observed, with Breztri showing higher incidence than Trelegy Ellipta, likely due to its more complex device usage. These findings highlight the need for enhanced patient education by healthcare providers to ensure proper device use in COPD treatment.

## 1. Introduction

Chronic obstructive pulmonary disease (COPD) is a common heterogeneous condition characterized by an abnormal inflammatory response of the lungs to noxious particles and gases.^[1]^ While COPD is primarily a neutrophilic disease, approximately 40% of COPD patients also exhibit eosinophilic airway inflammation,^[1]^ which can lead to progressive small airway obstruction, parenchymal destruction, and lung function decline. Chronic bronchitis and emphysema are the 2 main subtypes of COPD.^[2]^ COPD is associated with a high disease burden. The World Health Organization predicts that by 2030, it will rank as the third leading cause of death.^[3]^

For decades, inhaled bronchodilators such as β2-agonists and anticholinergic agents have been established as cornerstone treatments for COPD.^[4–6]^ These therapies alleviate and prevent symptoms, improve lung function, reduce dyspnea, and enhance health status.^[7,8]^ For patients with frequent exacerbations, inhaled corticosteroids (ICS) are primarily recommended.^[9]^ Due to their localized application, the safety concerns of these therapies are often underestimated by healthcare professionals and patients. With the rising prevalence of COPD in the elderly population, these patients are increasingly subject to polypharmacy.^[10]^ The use of multiple medications elevates the risk of drug-drug interactions, thereby increasing the incidence of adverse drug reactions (ADRs). Most available safety data on COPD treatments focus on the correct usage of inhalation devices.^[11,12]^

Budesonide/glycopyrrolate/formoterol (Breztri) and fluticasone furoate/umeclidinium/vilanterol (Trelegy Ellipta) are newly marketed fixed-dose triple bronchodilators for inhalation via a single device. The 2021 Global Initiative for Chronic Obstructive Lung Disease (GOLD) guidelines recommend both medications as first-line treatments for stable COPD patients.^[13]^ Breztri is a long-acting inhalation aerosol comprising budesonide, glycopyrronium bromide, and formoterol fumarate. This formulation leverages the advantages of all 3 components, offering synergistic effects that enhance their individual therapeutic actions.^[14]^ Trelegy Ellipta, also recommended in the GOLD guidelines,^[15]^ is a commonly used maintenance therapy for COPD. It combines an ICS, a long-acting β2-agonist, and a long-acting muscarinic antagonist (LAMA). Despite similarities in their mechanisms of action, the 2 drugs differ in their pharmacological properties and adverse reaction profiles.

Beyond efficacy, safety is a critical factor influencing medication choice, directly affecting clinical outcomes for patients. Current safety evaluations are primarily derived from clinical trials and individual case reports of adverse reactions. However, clinical trials often involve limited sample sizes, short follow-up durations, patient attrition, and stringent inclusion and exclusion criteria, which may not reflect real-world medication use. Therefore, post-marketing analyses of adverse event reports (AERs) in real-world settings are valuable for evaluating drug safety.^[16,17]^

Data mining techniques, such as signal detection algorithms, are increasingly used to explore medical databases and analyze large-scale data to identify potential associations between drugs and adverse reactions that may not have been observed in clinical trials.^[18]^ The FDA Adverse Event Reporting System (FAERS) is a public database designed to support the FDA’s post-marketing safety surveillance of drugs and therapeutic products. As one of the largest pharmacovigilance databases globally,^[19,20]^ FAERS data are publicly available and widely utilized by the FDA, healthcare systems, clinical researchers, and pharmaceutical companies to identify potential safety signals. Multinational pharmacovigilance analysis, integrating spontaneous reports across diverse regions and countries, enables a more comprehensive drug safety assessment by identifying potential signals undetected in single databases and revealing ADR heterogeneity across populations. Variability in regional prescribing practices and patient baseline characteristics significantly influence ADR incidence and profiles, thus underscoring the critical need for multi-database signal detection in effective pharmacovigilance. Therefore, it is of great significance to perform a multi-database analysis (e.g., Japanese Adverse Drug Event Report (JADER) database and Canada Vigilance Adverse Reaction (CVAR) database) to provide a more comprehensive evaluation of drug safety.

However, previous pharmacovigilance studies on Breztri and Trelegy Ellipta have primarily relied on single databases or clinical trials, lacking systematic multi-database comparisons and comprehensive signal detection using complementary analytical methods. To address this gap, this study conducted a multi-database disproportionality analysis integrating 4 algorithms to provide a more robust safety profile of the 2 drugs and to explore geographic heterogeneity in adverse event patterns. This study focused on mining the risk signals of adverse events associated with the use of these 2 drugs in the treatment of COPD. The objective was to provide further evidence and reference for their safe clinical application, offering insights for hospital decision-makers in drug selection and promoting rational clinical use of medications.

## 2. Methods and materials

### 2.1. Data source

The data for this study were obtained from the FAERS database, JADER database, CVAR database, covering 80 quarters of ASCII data packages from Q3 2004 to Q2 2024. Data cleaning and analysis were performed using R software version 4.4.0 and MySQL 8.0.

Given that this study utilized only de-identified, publicly available databases, it did not require approval from an ethics committee or institutional review board. Furthermore, the research did not necessitate informed consent from individual participants.

### 2.2. Data processing

The inclusion criteria were as follows: reports with a primary suspect (PS) drug corresponding to the study drugs (Breztri or Trelegy Ellipta) based on the specified search keywords, human reports, and reports with complete information on drug name and adverse event. Reports were excluded if they were duplicate records, if the drug was listed as a concomitant or interacting agent rather than the PS, or if the reported adverse event lacked a PT or a System Organ Class (SOC) mapped to Medical Dictionary for Regulatory Activities (MedDRA). The field “drugname” represents the drug name, and the field “PROD_AI” represents the product ingredients. The following terms were used as search keywords to identify ADR reports where the PS drug was Breztri and Trelegy Ellipta: “Breztri,” “breztriaerosphere,” “breztri aerosphere,” “budesonide glycopyrrolate formoterol,”’trelegyellipta’, “trelegyellipta,” “Trelegy Ellipta,” “fluticasone furoate umeclidinium vilanterol.”

Duplicate reports were removed following FDA-recommended methods. Reports were considered duplicates if they shared the same patient identification number, report date, and drug. When “case-id” and “fad-dt” were identical, the record with the higher “primary-id” was retained. If only “case-id” matched, the most recent “fad-dt” was selected to ensure unique reporting. PTs and SOC were harmonized using the MedDRA 26.1 dictionary. This process resulted in 394 ADR reports for Breztri and 18,866 ADR reports for TRELEGY ELLIPTA. Basic information included in the ADR reports was extracted to calculate safety warning signals. The study adhered to the disproportionality analysis methods described in the PharmacoVigilance (READUS-PV) guidelines for drug safety signal detection using individual case safety reports^[21]^ (Supplementary Table 1).

### 2.3. Descriptive analysis

Descriptive analysis was performed for baseline characteristics, including patient gender, age group, weight, reporter type, and reporting country. Additionally, the frequency and types of concomitant medications used with Breztri and Trelegy Ellipta were analyzed to explore potential drug-drug interactions. Categorical data were presented as counts (N) and percentages (%).

### 2.4. Onset time analysis

An onset time analysis was conducted to examine the temporal dynamics of ADRs. Reported ADRs were categorized into the following time intervals: 0 to 30 days, 31 to 60 days, 61 to 90 days, 91 to 120 days, 121 to 150 days, 151 to 180 days, 181 to 360 days, and beyond 360 days post-treatment. This analysis provided insights into the temporal patterns of ADR onset, critical for understanding the risk profiles associated with each drug.

### 2.5. Analytical methods

Disproportionality analysis methods are widely used for safety signal detection in healthcare databases. These methods calculate the observed-to-expected ratio of drug-event pairs based on a contingency table (Table 1). A disproportionality threshold indicates potential risk signals. This study selected the reporting odds ratio (ROR), proportional reporting ratio (PRR), Bayesian confidence propagation neural network (BCPNN), and multi-item gamma poisson shrinker (MGPS) methods, as these are the most commonly used approaches in pharmacovigilance, offering complementary strengths: frequentist methods (ROR and PRR) provide straightforward interpretability, while Bayesian methods (BCPNN and MGPS) offer greater stability in the presence of small sample sizes and reduce the impact of random variation. The detection criteria were set to balance sensitivity and specificity based on established practices in signal detection research. The detection criteria were as follows: Signal threshold: a ≥ 3; For ROR: The lower bound of the 95% confidence interval (CI) > 1; For PRR: The lower bound of the 95% CI > 1; For Bayesian analysis: information component (IC) lower limit (IC025) > 0 or Empirical Bayes Geometric Mean (EBGM05) > 2. {Fei, 2012 #59}{代菲, 2012 #60}^[22]^ Further details are provided in Table 2.

**Table 1:** A 4-cell table for disproportionality analysis method.

Drug	Target ADR Reports	Other ADR Reports	Total
Target drug	A	B	a + b
Other drugs	C	D	c + d
Total	a + c	b + d	a + b + c + d

ADR = adverse drug reaction.

**Table 2:** Formulas for disproportionality analysis method.

Methods	Formula	Threshold
ROR	$ROR\;\;\;=\;\;\;\frac{a/c}{b/d}$ $SE(lnROR)\;\;\;=\sqrt{\frac{1}{a}+\frac{1}{b}+\frac{1}{c}+\frac{1}{b}}$ $95\;\%\;CI\;\;\;=\;\;\;e^{ln(ROR)\;\pm \;1\mathrm{.96}se}$	a ≥ 3 ROR ≥ 3 95%CI > 1
PRR	$PRR\;\;\;=\;\;\;\frac{a(a+b)}{c(c+d)}$ $SE(lnPRR)=\sqrt{\frac{1}{a}\text{-}\frac{1}{a+b}+\frac{1}{c}\text{-}\frac{1}{c+b}}$ $95\;\%\;CI\;\;\;=e^{ln(PRR)\;\pm \;1\mathrm{.96}se}$ $x^{2}=\frac{{\left(ad\text{\textasciitilde{}}\text{-}bc\right)}^{2}\left(a+b+c+d\right)}{\left(a+b)(a+c)(c+d)(b+d\right)}$	a ≥ 3 PRR≥2 $x^{2}$≥4
BCPNN	$IC\;\;\;=\;\;\;log_{2}\frac{p(x,y)}{p(x)\;p(y)}\;\;\;=\;\;\;log_{2}\frac{a(a+b+c+d)}{\left(a+b)(a+c\right)}E(IC)$ $=\;\;\;log_{2}\frac{(a+\;\gamma \;11)(a+b+c+d+\;\alpha \;)(a+b+c+d+\;\beta \;)}{(a+b+c+d+\;\gamma \;)(a+b+\;\alpha \;1)(a+c+\;\beta \;1)}$ $\begin{array}{c} @l@l@l@l@l@l@l@l@l@l@l@l@l@l@l@l@l@l@l@l@V(IC)\;\;\;=\;\;\;\frac{1}{{\left(ln2\right)}^{2}}\;\;\;\{\;\left[\frac{(a+b+c+d)\text{\textasciitilde{}}\text{-}a+\;\gamma \;\text{-}\;\gamma \;11}{(a+\;\gamma \;11)(1+a+b+c+d+\;\gamma \;)}\right] \end{array}+$ $\begin{array}{c} @l@l@l@l@l@l@l@l@l@l@l@l@l@l@l@l@l@l@l@l@\left[\frac{(a+b+c+d)\text{\textasciitilde{}}\text{-}(a+b)+a\text{-}\;\alpha \;1}{(a+b+\;\alpha \;1)(1+a+b+c+d+\;\alpha \;)}\right]+ \end{array}\left[\frac{(a+b+c+d)\text{\textasciitilde{}}\text{-}(a+c)+\;\beta \;\text{-}\;\beta \;1}{(a+c+\;\beta \;1)(1+a+b+c+d+\;\beta \;)}\right]\}$ $\gamma \;\;=\;\;\;\gamma \;11\frac{(a+b+c+d+\;\alpha \;)+(a+b+c+d+\;\beta \;)}{(a+b+\;\alpha \;1)(a+c+\;\beta \;1)}$ $IC\text{-}2SD\;\;\;=\;\;\;E(IC)\text{-}2\sqrt{V(IC)}$	IC025 > 0
EBGM	$EBGM\;\;\;=\;\;\;\frac{a(a+b+c+d)}{\left(a+c)(a+b\right)}$ $SE(lnEBGM)\;\;\;=\sqrt{\frac{1}{a}+\frac{1}{b}+\frac{1}{c}+\frac{1}{b}}$ $95\;\%\;CI\;\;\;=\;\;\;e^{ln(EBGM)\;\pm \;1\mathrm{.96}se}$	EBGM05 > 2

BCPNN = Bayesian confidence propagation neural network, CI = confidence interval, EBGM = Empirical Bayes Geometric Mean, IC = information component, PRR = proportional reporting ratio, ROR = reporting odds ratio.

## 3. Results

### 3.1. Basic information

**Figure 1.**
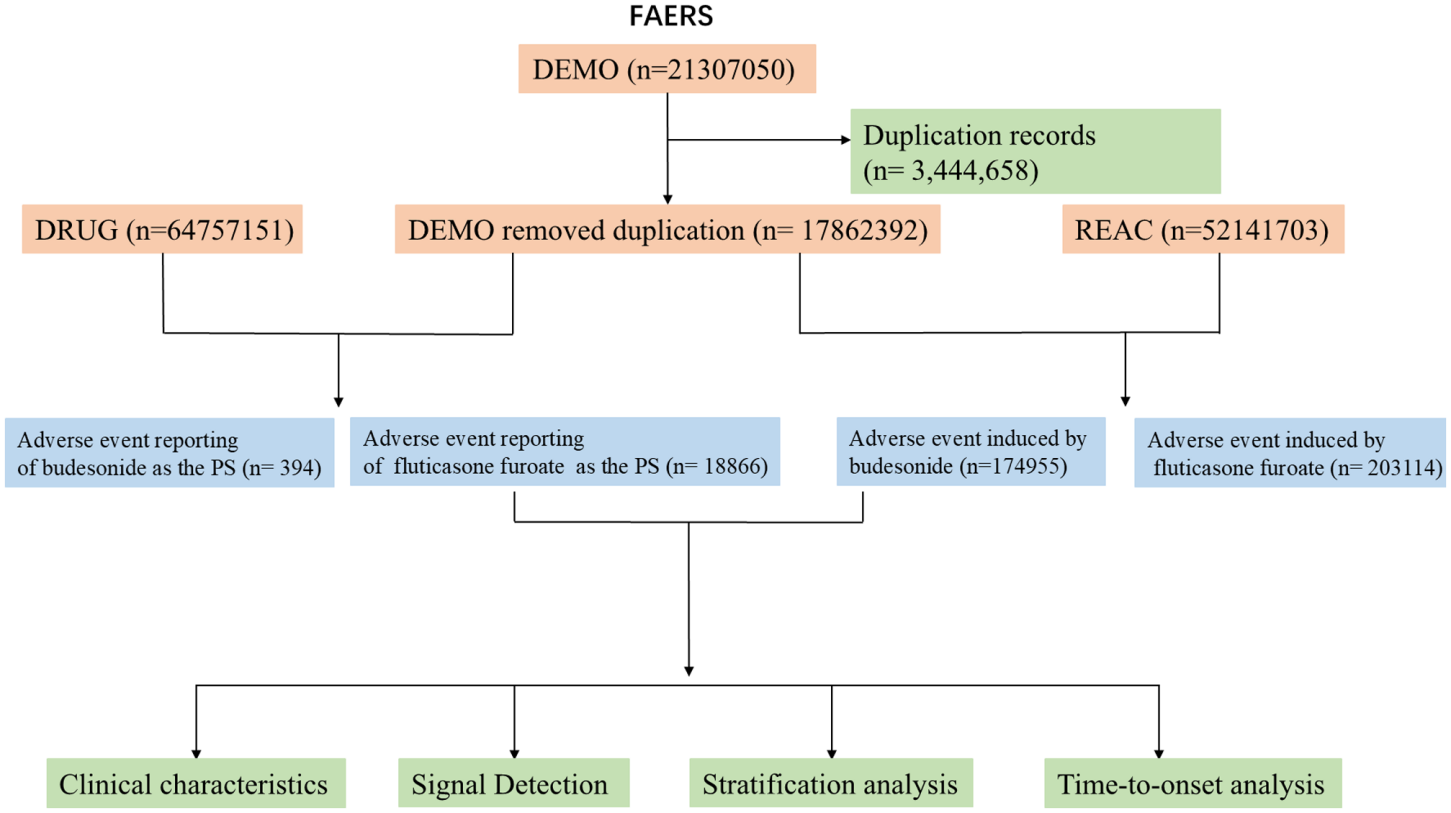
Flowchart for Breztri and Trelegy Ellipta

From Q3 2004 to Q2 2024, 19,260 AERs with Breztri or Trelegy Ellipta as the PS drugs were collected. These included 394 AERs for Breztri and 18,866 AERs for Trelegy Ellipta, as detailed in the flowchart (Fig. 1). After excluding abnormal or missing values, the number of AERs involving females exceeded that of males for both drugs. For Breztri, the reports included 123 males (31.2%), 212 females (53.8%), and 59 with missing gender information (15.0%). For Trelegy Ellipta, the reports included 6149 males (32.6%), 8924 females (47.3%), and 3793 with missing gender information (20.1%). The majority of patients were aged 65–85 years, with a body weight between 50–100 kg. The onset of adverse events was predominantly within 0–30 days of treatment initiation. The primary reporting source for both drugs was the United States, accounting for over 80% of reports. The majority of reporters were consumers (CN), followed by physicians (MD). The reported patient outcomes were predominantly classified as “Other health professional” (OT). Detailed data are provided in Table 3.

**Table 3:** Basic information on adverse drug reactions in FAERS.

	Breztri		Fluticasone furoate/umeclidinium/vilanterol (Trelegy Ellipta)	
Total number of adverse events	(N = 394)		(N = 18,866)	
Age	>85 years	6 (1.5%)	<18 years	17 (0.1%)
	18~64.9 years	37 (9.4%)	>85 years	367 (1.9%)
	65~85 years	56 (14.2%)	18~64.9 years	1027 (5.4%)
	Missing	295 (74.9%)	65~85 years	3055 (16.2%)
			Missing	14,400 (76.3%)
Gender	Female	212 (53.8%)	Female	8924 (47.3%)
	Male	123 (31.2%)	Male	6149 (32.6%)
	Missing	59 (15.0%)	Missing	3793 (20.1%)
Weight	<50 kg	8 (2.0%)	<50 kg	35 (0.2%)
	>100 kg	26 (6.6%)	>100 kg	91 (0.5%)
	50–100 kg	115 (29.2%)	50–100 kg	390 (2.1%)
	Missing	245 (62.2%)	Missing	18,350 (97.3%)
Report source	CN	224 (56.9%)	CN	16,413 (87.0%)
	HP	22 (5.6%)	HP	724 (3.8%)
	MD	78 (19.8%)	MD	1101 (5.8%)
	PH	3 (0.8%)	OT	138 (0.7%)
	Missing	67 (17.0%)	PH	463 (2.5%)
			Missing	27 (0.1%)
Reporting country	CNChina	8 (2.0%)	United states	16,004 (84.8%)
	United states	386 (98.0%)	Canada	1995 (10.6%)
			Japan	276 (1.5%)
			Great Britain	144 (0.8%)
Patient outcome		297 (75.4%)		12,693 (67.3%)
	Death	33 (8.4%)	Death	1725 (9.1%)
	Disability	2 (0.5%)	Disability	53 (0.3%)
	Hospitalization	22 (5.6%)	Hospitalization	2497 (13.2%)
	Life-Threatening	4 (1.0%)	Life-Threatening	51 (0.3%)
	Other	36 (9.1%)	Other	1845 (9.8%)
		297 (75.4%)		
Time to onset	0–30 days	10	0–30 days	432
	151–180 days	1	31–60 days	77
	61–90 days	3	61–90 days	45
			91–120 days	26
			121–150 days	23
			151–180 days	14
			181–360 days	94
			>360 days	209

N = counts, CN = Consumer, HP = Hospital Representative, MD = Medical Doctor, PH = Pharmacist, OT = Other.

### 3.2. Patterns of concomitant drug use

Table 4 summarizes the most commonly reported concomitant medications associated with Breztri and Trelegy Ellipta in the FAERS database. For Breztri, the most frequently reported concomitant drugs were Symbicort (118 reports), Spiriva (17 reports), and Albuterol (15 reports). For Trelegy Ellipta, the most common concomitant medications were Combivent (39 reports), Mucinex (16 reports), and Azithromycin anhydrous (7 reports).

**Table 4:** Top 3 most common concomitant medications in the FAERS database.

	Breztri (N)	Fluticasone furoate/umeclidinium/vilanterol (Trelegy Ellipta) (N)
Concomitant Medication 1	SYMBICORT (118)	COMBIVENT (39)
Concomitant Medication 2	SPIRIVA (17)	MUCINEX (16)
Concomitant Medication 3	ALBUTEROL (15)	AZITHROMYCIN ANHYDROUS (7)

N, counts.

### 3.3. Comparative analysis of SOCs for Breztri and Trelegy Ellipta

**Figure 2.**
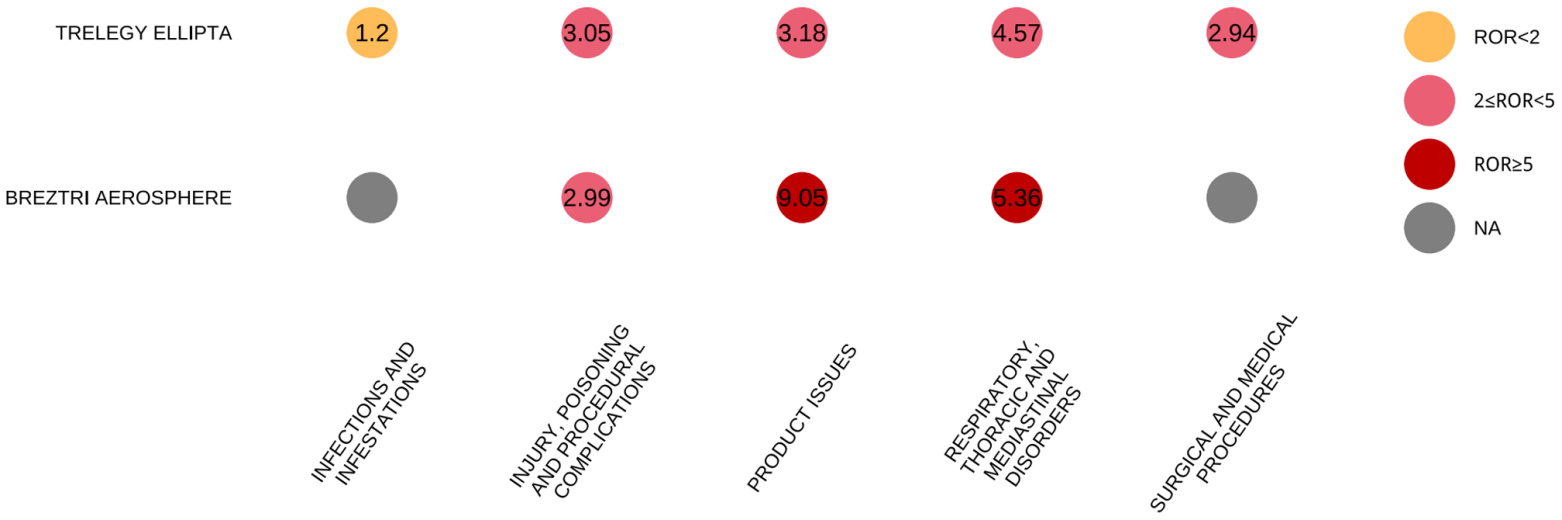
System organ classes (SOCs) involved in adverse drug reaction (ADR) signals caused by Breztri and Trelegy Ellipta.

The ADR signals for Breztri and Trelegy Ellipta were categorized by SOC. Positive signals with their respective SOCs were identified and statistically analyzed. For Breztri, 3 primary positive SOCs were identified: Product issues (ROR: 9.05, PRR: 8.04), respiratory, thoracic, and mediastinal disorders (ROR: 5.36, PRR: 4.44), and injury, poisoning, and procedural complications (ROR: 2.99, PRR: 2.52). For Trelegy Ellipta, 5 primary positive SOCs were identified: Respiratory, thoracic and mediastinal disorders (ROR: 4.57, PRR: 3.9), Product issues (ROR: 3.18, PRR: 3.07), Injury, poisoning, and procedural complications (ROR: 3.05, PRR: 2.56), Surgical and medical procedures (ROR: 2.94, PRR 2.86), and Infections and infestations (ROR: 1.2, PRR: 1.19). Further details are provided in Tables 5–7 and Figure 2.

**Table 5:** Signal-positive SOCs related to budesonide and fluticasone furoate in FAERS.

SOC	a	ROR (95%Cl)	PRR (Chi-Square Value)	EBGM (EBGM05)	IC (IC025)
Breztri					
Product issues	162	9.05 (7.68–10.67)	8.04 (1013.94)	8.04 (7)	3.01 (1.34)
Respiratory, thoracic and mediastinal disorders	273	5.36 (4.69–6.13)	4.44 (763)	4.44 (3.97)	2.15 (0.48)
Injury, poisoning and procedural complications	301	2.99 (2.62–3.4)	2.52 (304.32)	2.52 (2.26)	1.33 (-0.33)
Fluticasone furoate/umeclidinium/vilanterol (Trelegy Ellipta)					
Respiratory, thoracic and mediastinal disorders	7396	4.57 (4.45–4.68)	3.9 (16,709.36)	3.89 (3.81)	1.96 (0.29)
Product issues	1910	3.18 (3.04–3.33)	3.07 (2708.88)	3.07 (2.95)	1.62
Injury, poisoning and procedural complications	9436	3.05 (2.98–3.12)	2.56 (9890.53)	2.56 (2.51)	1.36 (-0.31)
Surgical and medical procedures	1532	2.94 (2.79–3.09)	2.86 (1878.68)	2.86 (2.74)	1.52
Infections and infestations	2484	1.2 (1.15–1.25)	1.19 (76.02)	1.19 (1.15)	0.25

EBGM = Empirical Bayes Geometric Mean, IC = information component, PRR = proportional reporting ratio, ROR = reporting odds ratio, SOC = System Organ Class.

**Table 6:** Basic information on adverse drug reactions in JADER.

	Breztri		Trelegy Ellipta	
Total number of adverse events	(N = 131)		(N = 376)	
Age				
Gender	Female	28 (21.4%)	Female	93 (24.7%)
	Male	99 (75.6%)	Male	229 (60.9%)
	Missing	4 (3.1%)	Missing	54 (14.4%)
Weight	/		/	
Report source	CN	20 (15.3%)	CN	87 (23.1%)
	HP	17 (13.0%)	PH	4 (1.1%)
	MD	91 (69.5%)	PH, CN	46 (12.2%)
	PH	2 (1.5%)	MP	2 (0.5%)
	Missing	1 (0.8%)	MP, CN	1 (0.3%)
			MD	35 (9.3%)
			MD, CN	188 (50.0%)
			MD, PH	1 (0.3%)
			MD, PH, CN	11 (2.9%)
			MD, MP, CN	1 (0.3%)
Reporting country	/		/	
Patient outcome	(N = 176)		(N = 527)	
	Sequelae	1 (0.6%)	Sequelae	6 (1.1%)
	Rehabilitation	40 (22.7%)	Rehabilitation	175 (33.2%)
	Mild rehabilitation	23 (13.1%)	Mild rehabilitation	67 (12.7%)
	Death	29 (16.5%)	Death	38 (7.2%)
	Not yet recovered	12 (6.8%)	Not yet recovered	13 (2.5%)
	Unknown	71 (40.3%)	Unknown	228 (43.3%)

N = counts, CN = Consumer, HP = Hospital Representative, MD = Medical Doctor, PH = Pharmacist, MP = Medical Professional.

**Table 7:** Basic information on adverse drug reactions in CAVR.

	Breztri		Trelegy Ellipta	
Total number of adverse events	(N = 31)		(N = 374)	
Age	>85	1 (3.2%)	>85	40 (10.7%)
	18~64.9	3 (9.7%)	18~64.9	66 (17.6%)
	65~85	9 (29.0%)	65~85	220 (58.8%)
	Missing	18 (58.1%)	Missing	48 (12.8%)
Weight	≥100 kg	1 (3.2%)	<50 kg	2 (0.5%)
	50–60 kg	2 (6.5%)	≥100 kg	6 (1.6%)
	60–70 kg	1 (3.2%)	50–60 kg	2 (0.5%)
	90–100 kg	1 (3.2%)	60–70 kg	23 (6.1%)
	Missing	26 (83.9%)	70–80 kg	8 (2.1%)
			80–90 kg	6 (1.6%)
			90–100 kg	4 (1.1%)
			Missing	323 (86.4%)
Report source	Consumer/other non health professional	10 (32.3%)	Consumer/other non health professional	219 (58.6%)
	Other health professional	5 (16.1%)	Other health professional	81 (21.7%)
	Pharmacist	2 (6.5%)	Pharmacist	29 (7.8%)
	Physician	2 (6.5%)	Physician	42 (11.2%)
	Missing	12 (38.7%)	Missing	3 (0.8%)
Reporting country				
Patient outcome	Recovered/resolved	6 (19.4%)	Fatal	140 (37.4%)
	Recovering/resolving	1 (3.2%)	Not recovered/not resolved	16 (4.3%)
	Unknown	24 (77.4%)	Recovered/resolved	41 (11.0%)
			Recovering/resolving	7 (1.9%)
			Unknown	170 (45.5%)

Note: N, counts.

### 3.4. Safety signals for Breztri and Trelegy Ellipta at the PT level in the FAERS database

**Figure 3.**
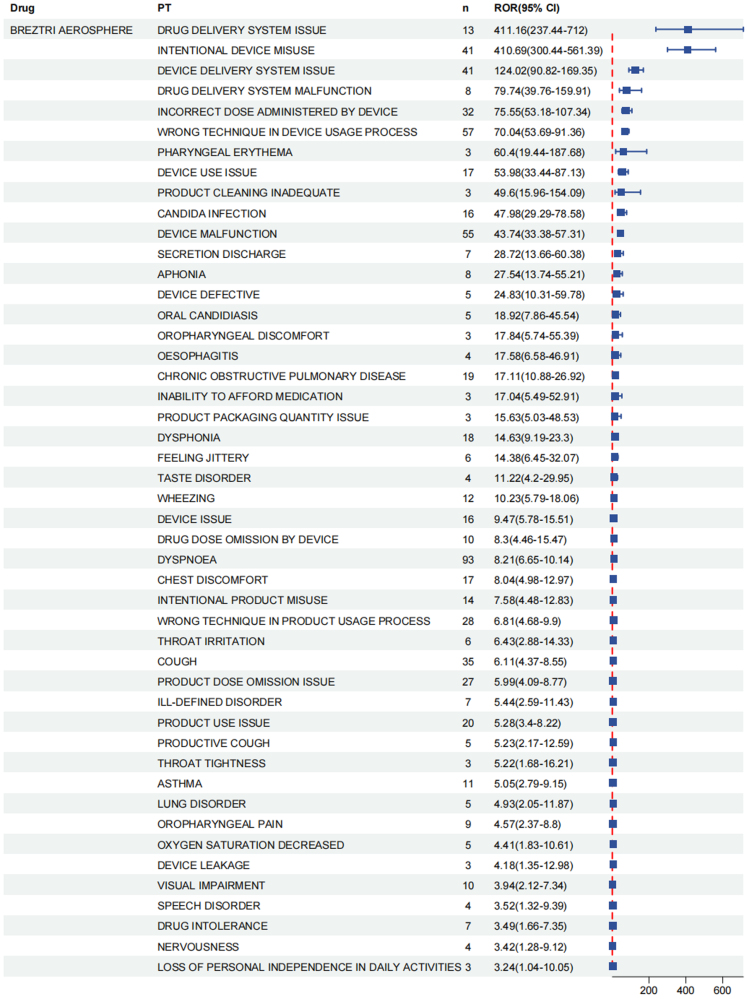
PTs associated with ADR signals of FAERS caused by Breztri. ADR = adverse drug reaction, FAERS = FDA Adverse Event Reporting System.

**Figure 4.**
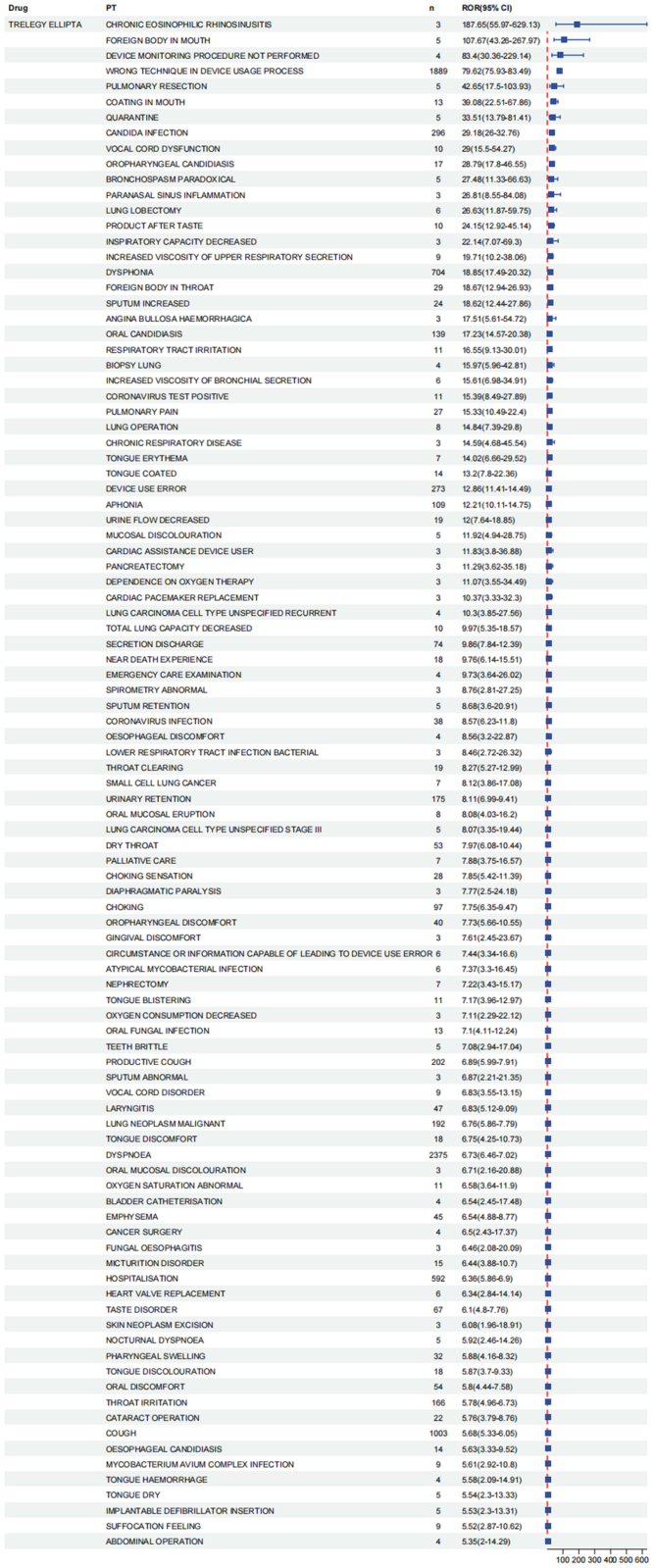
PTs associated with ADR signals of FAERS caused by Trelegy Ellipta. ADR = adverse drug reaction, FAERS = FDA Adverse Event Reporting System.

When calculating ADR signals for Breztri and Trelegy Ellipta, signals unrelated to the drugs were excluded. The top-ranked ADR signals and PTs for each drug were identified based on signal strength. Breztri had 47 PTs involving 11 SOCs, while Trelegy Ellipta had 160 PTs involving 15 SOCs. For Breztri, the top 10 ADR signals and their corresponding PTs were: Drug delivery system issue (ROR: 411.16, PRR: 407.01), Intentional device misuse (ROR: 410.69, PRR: 397.62), Device delivery system issue (ROR: 124.02, PRR: 120.09), Drug delivery system malfunction (ROR: 79.74, PRR 79.25), Incorrect dose administered by device (ROR: 75.55, PRR: 73.7), Wrong technique in device usage process (ROR: 70.04, PRR: 66.98), Pharyngeal erythema (ROR: 60.4, PRR 60.26), Device use issue (ROR: 53.98, PRR: 53.27), Product cleaning inadequate (ROR: 49.6, PRR: 49.48), and Candida infection (ROR: 47.98, PRR 47.39). Details are presented in Table S1 and Figure 3. For Trelegy Ellipta, the top 10 ADR signals and their corresponding PTs were: Chronic eosinophilic rhinosinusitis (ROR: 187.65, PRR: 187.63), Foreign body in mouth (ROR: 107.67, PRR: 107.66), Device monitoring procedure not performed (ROR: 83.4, PRR: 83.39), Wrong technique in device usage process (ROR: 79.62, PRR 75.88), Pulmonary resection (ROR: 42.65, PRR: 42.64), Coating in mouth (ROR: 39.08, PRR: 39.07), Quarantine (ROR: 33.51, PRR: 33.51), Candida infection (ROR: 29.18, PRR: 28.97), Vocal cord dysfunction (ROR: 29, PRR: 28.99), and Oropharyngeal candidiasis (ROR: 28.79, PRR: 28.77). Details are illustrated in Table S2 and Figure 4.

### 3.5. Safety signals for Breztri and Trelegy Ellipta at the PT level in the JADER database

**Figure 5.**
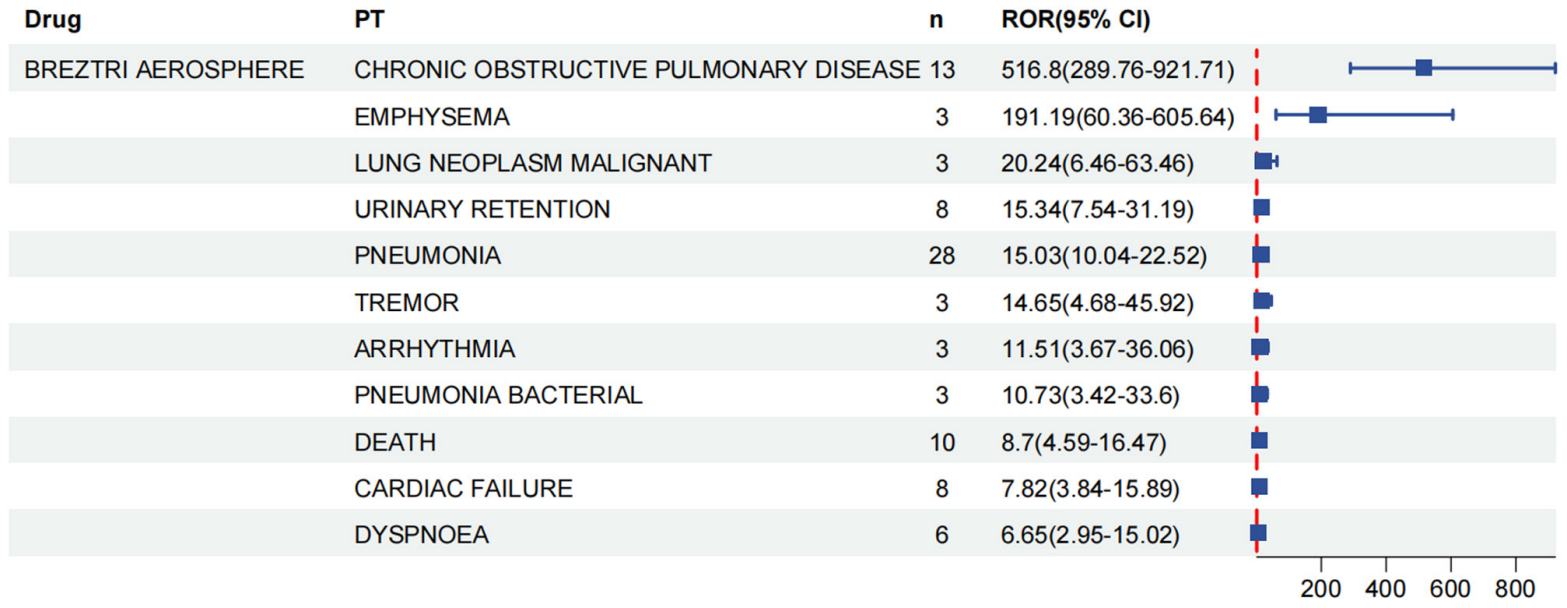
PTs associated with ADR signals of JADER database signals caused by Breztri. ADR = adverse drug reaction, JADER = Japanese Adverse Drug Event Report.

**Figure 6.**
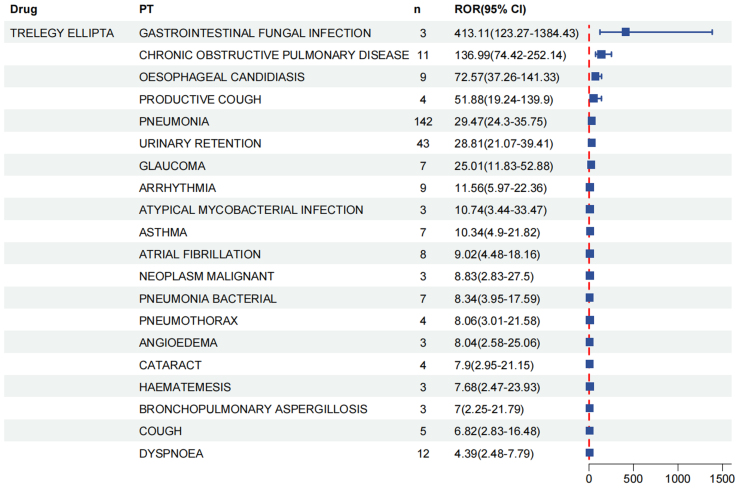
PTs associated with ADR signals of JADER database signals caused by Trelegy Ellipta. ADR = adverse drug reaction, JADER = Japanese Adverse Drug Event Report.

In the JADER database, the strongest ADR signals for Breztri were COPD (ROR: 516.8, PRR: 478.7), emphysema (ROR: 191.19, PRR: 187.95), and lung neoplasm malignant (ROR: 20.24, PRR: 19.92). Additional significant signals included urinary retention (ROR: 15.34, PRR: 14.68), pneumonia (ROR: 15.03, PRR: 12.8), tremor (ROR: 14.65, PRR: 14.42), arrhythmia (ROR: 11.51, PRR: 11.33), pneumonia bacterial (ROR: 10.73, PRR: 10.56), death (ROR: 8.70, PRR: 8.26), cardiac failure (ROR: 7.82, PRR: 7.51), and dyspnea (ROR: 6.65, PRR: 6.46) (Table S3 and Figure. 5). As shown in Table S4 and Figure. 6, analysis of Trelegy Ellipta in JADER identified gastrointestinal fungal infection as the top ADR signal (ROR: 413.11, PRR: 410.76), followed by COPD (ROR: 136.99, PRR: 134.15) and esophageal candidiasis (ROR: 72.57, PRR: 71.34). Other prominent signals were productive cough (ROR: 51.88, PRR: 51.49), pneumonia (ROR: 29.47, PRR: 21.8), urinary retention (ROR: 28.81, 26.54), glaucoma (ROR: 25.01, PRR: 24.69), arrhythmia (ROR: 11.56, PRR: 11.38), atypical mycobacterial infection (ROR: 10.74, PRR: 10.68), and asthma (ROR: 10.34, PRR: 10.22).

### 3.6. Safety signals for Breztri and Trelegy Ellipta at the PT level in the CAVR database

**Figure 7.**
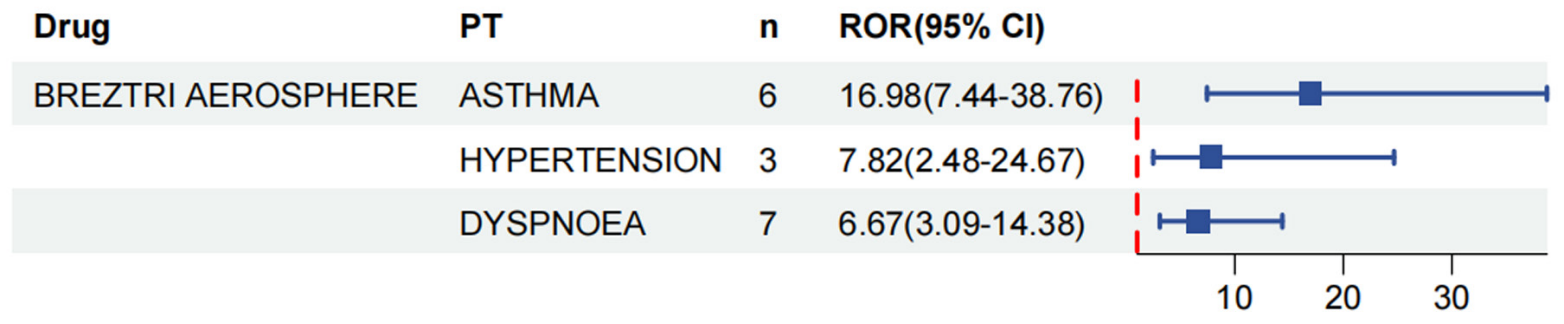
PTs associated with ADR signals of CAVR database signals caused by Breztri. ADR = adverse drug reaction.

**Figure 8.**
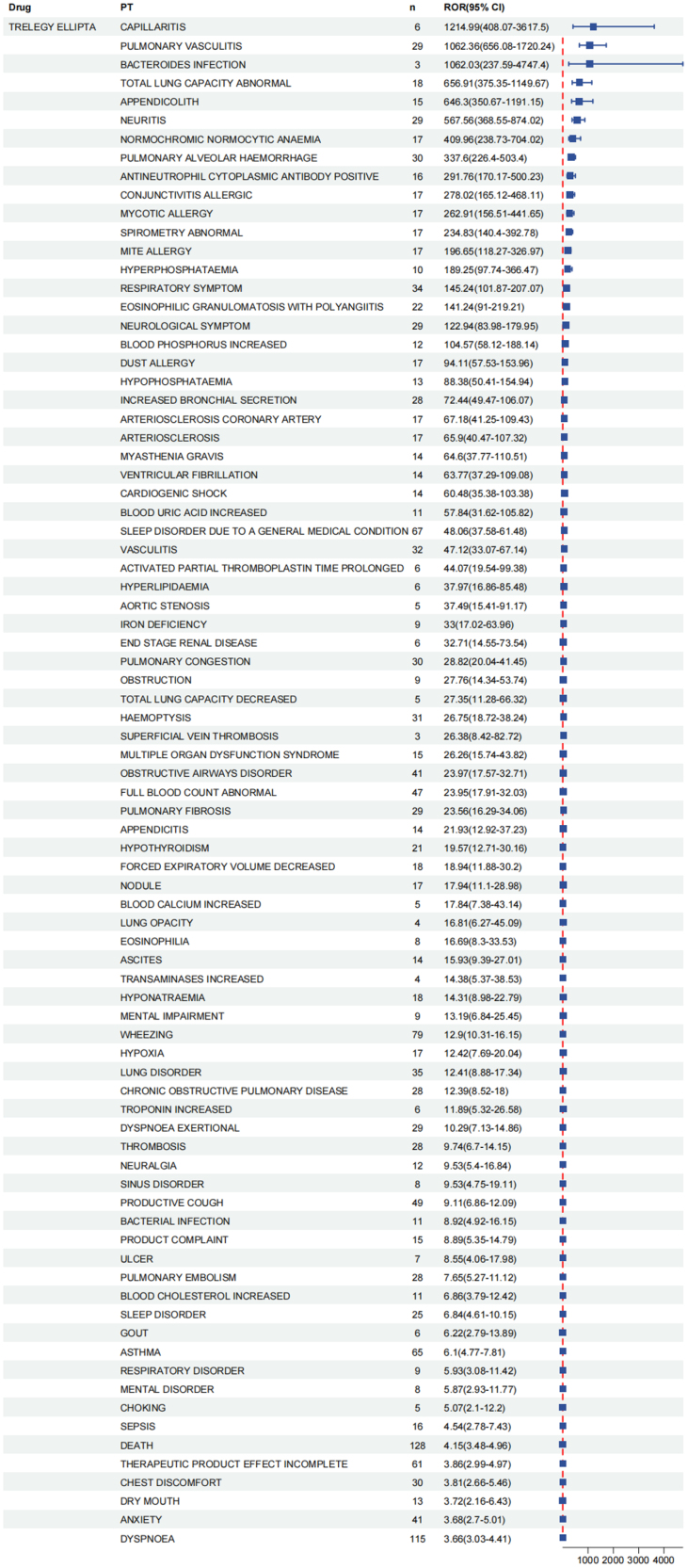
PTs associated with ADR signals of CAVR database signals caused by Trelegy Ellipta. ADR = adverse drug reaction.

Within the Canada Vigilance Adverse Reaction (CVAR) database, Breztri demonstrated significant ADR signals for asthma (ROR: 16.98, PRR: 16.03), hypertension (ROR: 7.82, PRR: 7.62), and dyspnea (ROR: 6.67, PRR: 6.28), with all signals showing robust risk associations (Table S5 and Fig. 7). For Trelegy Ellipta in CVAR database, the top ADR signals included capillaritis (ROR: 1214.99, PRR: 1212.51), pulmonary vasculitis (ROR: 1062.36, PRR: 1051.88), bacteriores infection (ROR: 1062.03, PRR: 1051.88), total lung capacity abnormal (ROR: 656.91, PRR: 652.89), appendicolith (ROR: 646.3, PRR: 643), neuritis (n = 29, ROR = 567.56; 95% CI: 368.55–874.02), normachromic normocytic anemia (ROR: 409.96, PRR: 407.6), pulmonary alveolar proteinosis (ROR: 337.6, PRR: 334.16), and antineutrophil cytoplasmic antibody positivity (ROR: 291.76, PRR: 290.17) (Table S6 and Fig. 8).

## 4. Discussion

COPD affects over 400 million individuals worldwide, with staggering costs. The World Economic Forum estimates that by 2030, the global economic burden of COPD will reach $50 trillion annually, surpassing cardiovascular diseases in expense.^[23]^ For patients with stable COPD, whose symptoms are relatively stable, pharmacological treatment is the primary therapeutic strategy, complementing education and counseling. Common medications for COPD include LABAs, LAMAs, and ICS.^[24]^ Trelegy Ellipta, a triple therapy formulation combining ICS, LAMA, and LABA, is widely used in clinical practice to alleviate symptoms in stable COPD patients. These components collectively exert anti-inflammatory and bronchodilatory effects, significantly reducing hospitalization rates and mortality in COPD patients.^[25]^ The common adverse reactions of Breztri and Trelegy Ellipta are similar, including nausea, cough, dysphonia, muscle cramps, palpitations, and oral candidiasis, most of which resolve spontaneously without intervention after discontinuation of the drug. However, Trelegy Ellipta is also commonly associated with nasopharyngitis and upper respiratory tract infections. The FULFIL study indicated that Trelegy Ellipta was associated with a higher incidence of pneumonia among upper respiratory infections.^[26]^ Despite their similar mechanisms, the 2 drugs differ in their pharmacological properties and adverse reaction profiles. Adverse events related to inhalation devices were observed for both drugs, with Breztri potentially having a higher incidence due to its more complex usage steps requiring hand-mouth coordination.

This study demonstrated that both Breztri and Trelegy Ellipta were novel fixed-dose triple bronchodilator therapies for inhalation via a single device. For both drugs, the male-to-female ratio of reported ADRs exceeded 1:1.3, and the majority of cases occurred in individuals aged 65 to 85 years, consistent with findings from a previous COPD study.^[27]^ Main reporters were consumers, followed by pharmacists and physicians. While consumers directly experience and report drug-related adverse events, pharmacists and physicians focus on monitoring medication use and periodically assessing comorbidities in COPD patients,^[28]^ thereby identifying adverse events. In addition to unspecified COPD, Breztri is primarily used for asthma and dyspnea. Both drugs are recommended for long-term treatment of moderate-to-severe stable COPD in domestic and international guidelines. Breztri was most frequently co-administered with Symbicort (118 cases), while Trelegy Ellipta was most frequently co-administered with Combivent (39 cases). Regarding adverse event outcomes, Breztri showed the highest proportion of severe outcomes, including mortality, warranting greater clinical attention to its adverse reactions. For Trelegy Ellipta, severe adverse events predominantly resulted in hospitalization or prolonged hospital stays. These differences suggest that Breztri may carry a higher risk of serious adverse events. Trelegy Ellipta had the highest number of reports in 2022, whereas no adverse events were reported for Breztri after 2021. Notably, no adverse events for Breztri were reported in individuals under 18 years of age. The United States was the primary reporting country, likely because these drugs were approved earlier in the U.S. Differences in patient demographics may introduce confounding factors related to ethnicity, gender, and reporter preferences.^[29]^

This study identified 15 SOCs for Trelegy Ellipta and 11 SOCs for Breztri. Unique SOC signals associated with Trelegy Ellipta included “Hepatobiliary disorders,” “Surgical and medical procedures,” “Pregnancy, puerperium, and perinatal conditions,” and “Reproductive system and breast disorders.” Trelegy Ellipta demonstrated weaker signal strength in “Infections and infestations” and “Surgical and medical procedures” but stronger signals in “Injury, poisoning, and procedural complications.” Pharmacological treatment of COPD aims to alleviate symptoms, reduce exacerbation frequency and severity, and improve exercise tolerance and health status.^[30]^ Given that COPD predominantly affects elderly populations, even when physicians provide thorough instructions on inhaler use and confirm patients’ understanding, some COPD patients fail to use the devices correctly. This improper use can lead to suboptimal disease control over time.

Breztri exhibited unique signals in “Product issues” and “Respiratory, thoracic, and mediastinal disorders,” with strong signal strength. The “Product issues” signal may be related to the delivery method of Breztri inhalation aerosol.^[31]^ Errors in drug storage and administration highlight the need for patient education regarding both medication use and proper storage. Some COPD patients may modify their lifestyles to compensate for symptoms and often seek medical care only when their condition significantly deteriorates.^[32]^

Our current study demonstrated that common adverse reactions shared by Breztri and fluticasone furoate included device usage errors, candidiasis, inability to afford medication, dysphonia, oral candidiasis, COPD, product omissions, aphonia, secretory discharge, oral discomfort, expectorant cough, dyspnea, taste disturbances, throat irritation, coughing, wheezing, oropharyngeal pain, pulmonary disorders, pharyngeal erythema, reduced oxygen saturation, and chest discomfort. Improper device use may cause drug deposition in the oropharynx, leading to respiratory, thoracic, and mediastinal disorders. Proper maintenance of nebulizer devices, including cleaning, disinfection, and storage, is critical. Inappropriate or negligent use and poor maintenance of nebulizers can result in serious health hazards, hospital-acquired infections, disease transmission, and other adverse outcomes.^[33]^ For “Infections and infestations,” Trelegy Ellipta exhibited stronger signal strength compared to Breztri. However, Breztri showed stronger signals for “Candidiasis” and “Oral candidiasis.” These findings suggest that Breztri users may be at higher risk for candidiasis, necessitating clinical vigilance to prevent disease progression. For high-risk patients, Trelegy Ellipta may be considered as an alternative therapy.

Most safety data for COPD treatment focus on the correct use of inhalation devices.^[11,12]^ Adverse reactions specific to Breztri primarily involve drug delivery system issues, intentional device misuse, device delivery system problems, drug delivery system malfunctions, incorrect doses administered by devices, device usage issues, inadequate product cleaning, equipment malfunctions, and device failures. Adverse reactions unique to Trelegy Ellipta include product name confusion, drug-disease interactions noted in the prescribing information, chronic eosinophilic rhinosinusitis, product complaints, foreign bodies in the mouth, product design-related confusion, device use errors, total pulmonary resection, and unperformed device monitoring procedures. Potential adverse reactions related to these drugs are often overlooked, and real-world data on ADRs in COPD patients remain limited.^[34,35]^

Most adverse reactions are not caused by the inhalation therapy itself, nor are they attributable to patients or healthcare professionals. This phenomenon is well-documented, as the systemic effects of inhalation therapies have long been underestimated due to their mode of application. However, cardiovascular adverse reactions associated with inhaled β2-agonists may correlate with the high prevalence of cardiovascular events in COPD patients, a finding that is not new.^[36]^ Given the widespread prescription of inhaled medications, enhanced pharmacovigilance, patient education, and dissemination of information to patients are crucial to preventing unnecessary ADRs.

Cross-database analysis revealed both convergent and divergent adverse drug reaction (ADR) signals for budesonide/glycopyrronium/formoterol (Breztri) and Trelegy Ellipta. All 3 databases consistently identified respiratory risks as core safety concerns: COPD exacerbation was prominent in JADER (Breztri ROR = 516.8; Trelegy ROR = 136.99) and FAERS (Trelegy-associated chronic eosinophilic rhinosinusitis, ROR = 187.65), while CVARD highlighted obstructive airway disorders (Trelegy, n = 60) and dyspnea (Breztri ROR = 6.67). Pneumonia signals recurred across databases, particularly in JADER (Trelegy ROR = 29.47; Breztri ROR = 15.03) and FAERS (both drugs). Notable divergences emerged in infection patterns: JADER emphasized Trelegy-linked gastrointestinal fungal infections (ROR = 413.11) and esophageal candidiasis (ROR = 72.57), whereas FAERS prioritized Breztri-associated candidiasis (ROR = 47.98), suggesting regional susceptibility differences. Device-related signals dominated FAERS for both drugs (Breztri: delivery system issues, ROR > 400; Trelegy: technique errors, ROR = 79.62) but were absent in JADER and minimal in CVARD (Trelegy-associated lung capacity abnormalities, n = 30), indicating stricter device surveillance in Western databases. Cardiovascular/anticholinergic risks showed trans-database consistency, urinary retention (JADER: Breztri ROR = 15.34, Trelegy ROR = 28.81) and arrhythmia (JADER: both drugs), yet hypertension emerged only in CVARD (Breztri ROR = 7.82). Unique signals included Trelegy-associated vasculitis (CVARD: pulmonary vasculitis n = 29, ANCA positivity n = 17) and Breztri-linked lung malignancy in JADER (ROR = 20.24), highlighting region-specific vulnerabilities. FAERS exclusively captured human-factor risks (e.g., “intentional device misuse” ROR = 410.69 for Breztri), underscoring cultural disparities in reporting. These findings demonstrate that while core respiratory and anticholinergic risks are globally relevant, infection profiles, device complications, and rare immunological events exhibit significant geographic heterogeneity, necessitating tailored risk mitigation strategies aligned with regional pharmacovigilance patterns.

Several limitations should be acknowledged. First, the FAERS, JADER, and CVAR databases are spontaneous reporting systems subject to underreporting, incomplete data, and reporting biases, which may affect the accuracy and generalizability of signal detection. Second, disproportionality analysis identifies statistical associations but cannot establish causality; the observed signals should be interpreted as potential safety signals rather than confirmed adverse reactions. Third, despite standardization using MedDRA, cross-country differences in reporting practices, coding preferences, and regulatory requirements may introduce uncontrolled heterogeneity. Finally, the generalizability of findings is limited as most reports originated from the United States and Japan. Future studies using electronic health records or prospective cohort designs are warranted to validate the identified safety signals.

## 5. Conclusion

When prescribing Breztri and Trelegy Ellipta, clinicians should monitor not only ADR signals documented in the prescribing information but also potential new ADR signals, such as inadequate product cleaning, unperformed device monitoring procedures, and incorrect device usage. These new signals may arise from the complexity of device operation, which requires proper hand-mouth coordination. This underscores the critical role of physicians and pharmacists in providing comprehensive patient education on device use during COPD treatment. Although core respiratory and anticholinergic risks are globally relevant, infection profiles, device complications, and rare immunological events exhibit significant geographic heterogeneity, necessitating tailored risk mitigation strategies aligned with regional pharmacovigilance patterns.

## Author contributions

**Conceptualization:** Junyu Wang.

**Data curation:** Junyu Wang, Kexu Chen.

**Formal analysis:** Junyu Wang, Kexu Chen.

**Methodology:** Lu Yun.

**Supervision:** Kexu Chen.

**Software:** Lu Yun.

**Writing – original draft:** Junyu Wang.

**Writing – review & editing:** Junyu Wang, Kexu Chen, Lu Yun, Dexiang Xu.












