# Supplementary material for: Adverse event mining for Breztri and Trelegy Ellipta based on the three international pharmacovigilance databases
Source: Medicine (Baltimore). 2026 Jun 5;105(23):e49162. doi: 10.1097/MD.0000000000049162 (PMC13246110; doi:10.1097/MD.0000000000049162)
Supplement: Supplementary file 1 [file medi-105-e49162-s001.docx]

Table S1 Signal-positive ADE PTs for Breztri of FAERS

| soc_name_en | N | PT | a | ROR (95%Cl) | PRR (Chi-Square Value) | EBGM (EBGM05) | IC (IC025) |
| --- | --- | --- | --- | --- | --- | --- | --- |
| INJURY, POISONING AND PROCEDURAL COMPLICATIONS | 246 | WRONG TECHNIQUE IN DEVICE USAGE PROCESS | 57 | 70.04 (53.69 - 91.36) | 66.98 (3700.99) | 66.87 (53.54) | 6.06 (4.4) |
|  |  | INTENTIONAL DEVICE MISUSE | 41 | 410.69 (300.44 - 561.39) | 397.62 (16064.25) | 393.77 (303.15) | 8.62 (6.95) |
|  |  | INCORRECT DOSE ADMINISTERED BY DEVICE | 32 | 75.55 (53.18 - 107.34) | 73.7 (2291.3) | 73.56 (54.83) | 6.2 (4.53) |
|  |  | WRONG TECHNIQUE IN PRODUCT USAGE PROCESS | 28 | 6.81 (4.68 - 9.9) | 6.68 (135.74) | 6.68 (4.88) | 2.74 (1.07) |
|  |  | PRODUCT DOSE OMISSION ISSUE | 27 | 5.99 (4.09 - 8.77) | 5.89 (109.92) | 5.89 (4.28) | 2.56 (0.89) |
|  |  | PRODUCT USE ISSUE | 20 | 5.28 (3.4 - 8.22) | 5.22 (68.35) | 5.22 (3.6) | 2.38 (0.72) |
|  |  | DEVICE USE ISSUE | 17 | 53.98 (33.44 - 87.13) | 53.27 (871.06) | 53.21 (35.64) | 5.73 (4.07) |
|  |  | INTENTIONAL PRODUCT MISUSE | 14 | 7.58 (4.48 - 12.83) | 7.51 (79.06) | 7.51 (4.83) | 2.91 (1.24) |
|  |  | DRUG DOSE OMISSION BY DEVICE | 10 | 8.3 (4.46 - 15.47) | 8.25 (63.71) | 8.24 (4.9) | 3.04 (1.38) |
| RESPIRATORY, THORACIC AND MEDIASTINAL DISORDERS | 230 | DYSPNOEA | 93 | 8.21 (6.65 - 10.14) | 7.69 (546.44) | 7.69 (6.45) | 2.94 (1.28) |
|  |  | COUGH | 35 | 6.11 (4.37 - 8.55) | 5.97 (145.44) | 5.97 (4.51) | 2.58 (0.91) |
|  |  | CHRONIC OBSTRUCTIVE PULMONARY DISEASE | 19 | 17.11 (10.88 - 26.92) | 16.87 (283.8) | 16.86 (11.54) | 4.08 (2.41) |
|  |  | DYSPHONIA | 18 | 14.63 (9.19 - 23.3) | 14.44 (225.33) | 14.44 (9.78) | 3.85 (2.18) |
|  |  | WHEEZING | 12 | 10.23 (5.79 - 18.06) | 10.14 (98.97) | 10.14 (6.3) | 3.34 (1.67) |
|  |  | ASTHMA | 11 | 5.05 (2.79 - 9.15) | 5.02 (35.45) | 5.02 (3.05) | 2.33 (0.66) |
|  |  | OROPHARYNGEAL PAIN | 9 | 4.57 (2.37 - 8.8) | 4.54 (24.91) | 4.54 (2.62) | 2.18 (0.52) |
|  |  | APHONIA | 8 | 27.54 (13.74 - 55.21) | 27.38 (203.24) | 27.36 (15.29) | 4.77 (3.11) |
|  |  | THROAT IRRITATION | 6 | 6.43 (2.88 - 14.33) | 6.4 (27.36) | 6.4 (3.27) | 2.68 (1.01) |
|  |  | PRODUCTIVE COUGH | 5 | 5.23 (2.17 - 12.59) | 5.22 (17.05) | 5.22 (2.5) | 2.38 (0.71) |
|  |  | LUNG DISORDER | 5 | 4.93 (2.05 - 11.87) | 4.92 (15.61) | 4.92 (2.36) | 2.3 (0.63) |
|  |  | THROAT TIGHTNESS | 3 | 5.22 (1.68 - 16.21) | 5.21 (10.21) | 5.21 (2.02) | 2.38 (0.71) |
|  |  | PHARYNGEAL ERYTHEMA | 3 | 60.4 (19.44 - 187.68) | 60.26 (174.58) | 60.17 (23.3) | 5.91 (4.24) |
|  |  | OROPHARYNGEAL DISCOMFORT | 3 | 17.84 (5.74 - 55.39) | 17.8 (47.54) | 17.79 (6.89) | 4.15 (2.48) |
| PRODUCT ISSUES | 147 | DEVICE MALFUNCTION | 55 | 43.74 (33.38 - 57.31) | 41.91 (2196.41) | 41.87 (33.4) | 5.39 (3.72) |
|  |  | DEVICE DELIVERY SYSTEM ISSUE | 41 | 124.02 (90.82 - 169.35) | 120.09 (4829.08) | 119.74 (92.26) | 6.9 (5.24) |
|  |  | DEVICE ISSUE | 16 | 9.47 (5.78 - 15.51) | 9.37 (119.68) | 9.36 (6.2) | 3.23 (1.56) |
|  |  | DRUG DELIVERY SYSTEM ISSUE | 13 | 411.16 (237.44 - 712) | 407.01 (5213.06) | 402.98 (254.54) | 8.65 (6.98) |
|  |  | DRUG DELIVERY SYSTEM MALFUNCTION | 8 | 79.74 (39.76 - 159.91) | 79.25 (616.95) | 79.1 (44.19) | 6.31 (4.64) |
|  |  | DEVICE DEFECTIVE | 5 | 24.83 (10.31 - 59.78) | 24.74 (113.84) | 24.72 (11.85) | 4.63 (2.96) |
|  |  | PRODUCT CLEANING INADEQUATE | 3 | 49.6 (15.96 - 154.09) | 49.48 (142.34) | 49.42 (19.14) | 5.63 (3.96) |
|  |  | PRODUCT PACKAGING QUANTITY ISSUE | 3 | 15.63 (5.03 - 48.53) | 15.59 (40.97) | 15.59 (6.04) | 3.96 (2.29) |
|  |  | DEVICE LEAKAGE | 3 | 4.18 (1.35 - 12.98) | 4.17 (7.25) | 4.17 (1.62) | 2.06 (0.39) |
| GENERAL DISORDERS AND ADMINISTRATION SITE CONDITIONS | 44 | CHEST DISCOMFORT | 17 | 8.04 (4.98 - 12.97) | 7.94 (103.35) | 7.94 (5.32) | 2.99 (1.32) |
|  |  | DRUG INTOLERANCE | 7 | 3.49 (1.66 - 7.35) | 3.48 (12.4) | 3.48 (1.87) | 1.8 (0.13) |
|  |  | ILL-DEFINED DISORDER | 7 | 5.44 (2.59 - 11.43) | 5.41 (25.21) | 5.41 (2.91) | 2.44 (0.77) |
|  |  | SECRETION DISCHARGE | 7 | 28.72 (13.66 - 60.38) | 28.57 (186.13) | 28.55 (15.33) | 4.84 (3.17) |
|  |  | FEELING JITTERY | 6 | 14.38 (6.45 - 32.07) | 14.32 (74.32) | 14.31 (7.31) | 3.84 (2.17) |
| INFECTIONS AND INFESTATIONS | 21 | CANDIDA INFECTION | 16 | 47.98 (29.29 - 78.58) | 47.39 (725.95) | 47.34 (31.33) | 5.56 (3.9) |
|  |  | ORAL CANDIDIASIS | 5 | 18.92 (7.86 - 45.54) | 18.85 (84.49) | 18.84 (9.03) | 4.24 (2.57) |
| EYE DISORDERS | 10 | VISUAL IMPAIRMENT | 10 | 3.94 (2.12 - 7.34) | 3.92 (21.78) | 3.92 (2.33) | 1.97 (0.3) |
| NERVOUS SYSTEM DISORDERS | 8 | TASTE DISORDER | 4 | 11.22 (4.2 - 29.95) | 11.19 (37.12) | 11.19 (4.92) | 3.48 (1.82) |
|  |  | SPEECH DISORDER | 4 | 3.52 (1.32 - 9.39) | 3.51 (7.19) | 3.51 (1.54) | 1.81 (0.14) |
| SOCIAL CIRCUMSTANCES | 6 | LOSS OF PERSONAL INDEPENDENCE IN DAILY ACTIVITIES | 3 | 3.24 (1.04 - 10.05) | 3.23 (4.62) | 3.23 (1.25) | 1.69 (0.02) |
|  |  | INABILITY TO AFFORD MEDICATION | 3 | 17.04 (5.49 - 52.91) | 17 (45.17) | 16.99 (6.58) | 4.09 (2.42) |
| INVESTIGATIONS | 5 | OXYGEN SATURATION DECREASED | 5 | 4.41 (1.83 - 10.61) | 4.4 (13.13) | 4.4 (2.11) | 2.14 (0.47) |
| GASTROINTESTINAL DISORDERS | 4 | OESOPHAGITIS | 4 | 17.58 (6.58 - 46.91) | 17.52 (62.31) | 17.52 (7.7) | 4.13 (2.46) |
| PSYCHIATRIC DISORDERS | 4 | NERVOUSNESS | 4 | 3.42 (1.28 - 9.12) | 3.41 (6.81) | 3.41 (1.5) | 1.77 (0.1) |

Note: N, counts, ROR, reporting odds ratio; PRR, proportional reporting ratio; IC, information component; EBGM, Empirical Bayes Geometric Mean; PT, Preferred Term.
