# Supplementary material for: Adverse event mining for Breztri and Trelegy Ellipta based on the three international pharmacovigilance databases
Source: Medicine (Baltimore). 2026 Jun 5;105(23):e49162. doi: 10.1097/MD.0000000000049162 (PMC13246110; doi:10.1097/MD.0000000000049162)
Supplement: Supplementary file 2 [file medi-105-e49162-s002.docx]

Table S2 Signal-positive ADE PTs for Trelegy Ellipta of FAERS

| soc_name_en | N | PT | a | ROR(95%Cl) | PRR (Chi-Square Value) | EBGM (EBGM05) | IC (IC025) |
| --- | --- | --- | --- | --- | --- | --- | --- |
| RESPIRATORY, THORACIC AND MEDIASTINAL DISORDERS | 5826 | DYSPNOEA | 2375 | 6.73 (6.46 - 7.02) | 6.39 (10842.5) | 6.36 (6.14) | 2.67 (1) |
|  |  | COUGH | 1003 | 5.68 (5.33 - 6.05) | 5.56 (3752.39) | 5.54 (5.26) | 2.47 (0.8) |
|  |  | DYSPHONIA | 704 | 18.85 (17.49 - 20.32) | 18.53 (11526.67) | 18.29 (17.18) | 4.19 (2.53) |
|  |  | OROPHARYNGEAL PAIN | 263 | 4.33 (3.84 - 4.89) | 4.31 (667.77) | 4.3 (3.89) | 2.1 (0.44) |
|  |  | PRODUCTIVE COUGH | 202 | 6.89 (5.99 - 7.91) | 6.86 (1005.73) | 6.82 (6.08) | 2.77 (1.1) |
|  |  | WHEEZING | 171 | 4.71 (4.05 - 5.48) | 4.69 (495.82) | 4.68 (4.13) | 2.23 (0.56) |
|  |  | THROAT IRRITATION | 166 | 5.78 (4.96 - 6.73) | 5.76 (650) | 5.74 (5.05) | 2.52 (0.85) |
|  |  | LUNG DISORDER | 126 | 4.03 (3.38 - 4.8) | 4.02 (285.51) | 4.01 (3.47) | 2 (0.34) |
|  |  | APHONIA | 109 | 12.21 (10.11 - 14.75) | 12.18 (1108.42) | 12.08 (10.31) | 3.59 (1.93) |
|  |  | CHOKING | 97 | 7.75 (6.35 - 9.47) | 7.73 (565.68) | 7.7 (6.51) | 2.94 (1.28) |
|  |  | DYSPNOEA EXERTIONAL | 83 | 3.48 (2.81 - 4.32) | 3.48 (146.05) | 3.47 (2.9) | 1.79 (0.13) |
|  |  | DRY THROAT | 53 | 7.97 (6.08 - 10.44) | 7.96 (320.51) | 7.92 (6.31) | 2.98 (1.32) |
|  |  | RESPIRATORY TRACT CONGESTION | 46 | 4.5 (3.37 - 6.01) | 4.5 (124.68) | 4.48 (3.52) | 2.17 (0.5) |
|  |  | EMPHYSEMA | 45 | 6.54 (4.88 - 8.77) | 6.53 (209.93) | 6.51 (5.09) | 2.7 (1.04) |
|  |  | OROPHARYNGEAL DISCOMFORT | 40 | 7.73 (5.66 - 10.55) | 7.72 (232.75) | 7.68 (5.92) | 2.94 (1.28) |
|  |  | PNEUMOTHORAX | 36 | 3.48 (2.51 - 4.82) | 3.47 (63.3) | 3.47 (2.64) | 1.79 (0.13) |
|  |  | PHARYNGEAL SWELLING | 32 | 5.88 (4.16 - 8.32) | 5.88 (128.96) | 5.86 (4.38) | 2.55 (0.88) |
|  |  | CHOKING SENSATION | 28 | 7.85 (5.42 - 11.39) | 7.85 (166.38) | 7.81 (5.72) | 2.97 (1.3) |
|  |  | PULMONARY PAIN | 27 | 15.33 (10.49 - 22.4) | 15.32 (357.21) | 15.15 (11.03) | 3.92 (2.25) |
|  |  | SPUTUM INCREASED | 24 | 18.62 (12.44 - 27.86) | 18.61 (394.31) | 18.36 (13.11) | 4.2 (2.53) |
|  |  | SPUTUM DISCOLOURED | 22 | 3.58 (2.35 - 5.43) | 3.57 (40.69) | 3.57 (2.51) | 1.83 (0.17) |
|  |  | RESPIRATION ABNORMAL | 21 | 4.91 (3.2 - 7.54) | 4.91 (65.11) | 4.89 (3.42) | 2.29 (0.62) |
|  |  | THROAT CLEARING | 19 | 8.27 (5.27 - 12.99) | 8.27 (120.7) | 8.23 (5.64) | 3.04 (1.37) |
|  |  | INCREASED UPPER AIRWAY SECRETION | 17 | 4.69 (2.91 - 7.54) | 4.68 (49.09) | 4.67 (3.14) | 2.22 (0.56) |
|  |  | RESPIRATORY TRACT IRRITATION | 11 | 16.55 (9.13 - 30.01) | 16.55 (158.71) | 16.36 (9.94) | 4.03 (2.36) |
|  |  | VOCAL CORD DYSFUNCTION | 10 | 29 (15.5 - 54.27) | 28.99 (264.45) | 28.39 (16.8) | 4.83 (3.16) |
|  |  | VOCAL CORD DISORDER | 9 | 6.83 (3.55 - 13.15) | 6.83 (44.55) | 6.8 (3.93) | 2.77 (1.1) |
|  |  | SUFFOCATION FEELING | 9 | 5.52 (2.87 - 10.62) | 5.52 (33.14) | 5.5 (3.18) | 2.46 (0.79) |
|  |  | INCREASED VISCOSITY OF UPPER RESPIRATORY SECRETION | 9 | 19.71 (10.2 - 38.06) | 19.7 (157.41) | 19.42 (11.2) | 4.28 (2.61) |
|  |  | INCREASED BRONCHIAL SECRETION | 7 | 4.9 (2.33 - 10.3) | 4.9 (21.67) | 4.89 (2.63) | 2.29 (0.62) |
|  |  | INCREASED VISCOSITY OF BRONCHIAL SECRETION | 6 | 15.61 (6.98 - 34.91) | 15.6 (81.05) | 15.43 (7.87) | 3.95 (2.28) |
|  |  | BRONCHITIS CHRONIC | 6 | 3.56 (1.6 - 7.93) | 3.56 (11) | 3.55 (1.82) | 1.83 (0.16) |
|  |  | PHARYNGEAL ERYTHEMA | 6 | 3.91 (1.75 - 8.71) | 3.91 (12.94) | 3.9 (1.99) | 1.96 (0.3) |
|  |  | NOCTURNAL DYSPNOEA | 5 | 5.92 (2.46 - 14.26) | 5.92 (20.36) | 5.9 (2.83) | 2.56 (0.89) |
|  |  | BRONCHOSPASM PARADOXICAL | 5 | 27.48 (11.33 - 66.63) | 27.48 (124.96) | 26.93 (12.84) | 4.75 (3.08) |
|  |  | SPUTUM RETENTION | 5 | 8.68 (3.6 - 20.91) | 8.68 (33.73) | 8.62 (4.13) | 3.11 (1.44) |
|  |  | LOWER RESPIRATORY TRACT CONGESTION | 4 | 3.67 (1.37 - 9.78) | 3.67 (7.73) | 3.66 (1.61) | 1.87 (0.2) |
|  |  | OROPHARYNGEAL BLISTERING | 4 | 3.49 (1.31 - 9.31) | 3.49 (7.09) | 3.48 (1.53) | 1.8 (0.13) |
|  |  | PHARYNGEAL PARAESTHESIA | 3 | 3.37 (1.08 - 10.45) | 3.36 (4.97) | 3.36 (1.3) | 1.75 (0.08) |
|  |  | CHRONIC RESPIRATORY FAILURE | 3 | 4.05 (1.31 - 12.59) | 4.05 (6.88) | 4.04 (1.57) | 2.02 (0.35) |
|  |  | CHRONIC EOSINOPHILIC RHINOSINUSITIS | 3 | 187.65 (55.97 - 629.13) | 187.63 (487.3) | 164.3 (59.71) | 7.36 (5.6) |
|  |  | PHARYNGEAL HAEMORRHAGE | 3 | 3.67 (1.18 - 11.41) | 3.67 (5.82) | 3.66 (1.42) | 1.87 (0.21) |
|  |  | PARANASAL SINUS INFLAMMATION | 3 | 26.81 (8.55 - 84.08) | 26.8 (73.04) | 26.29 (10.1) | 4.72 (3.03) |
|  |  | DIAPHRAGMATIC PARALYSIS | 3 | 7.77 (2.5 - 24.18) | 7.77 (17.6) | 7.73 (2.99) | 2.95 (1.28) |
|  |  | CHRONIC RESPIRATORY DISEASE | 3 | 14.59 (4.68 - 45.54) | 14.59 (37.57) | 14.44 (5.57) | 3.85 (2.18) |
| INJURY, POISONING AND PROCEDURAL COMPLICATIONS | 2219 | WRONG TECHNIQUE IN DEVICE USAGE PROCESS | 1889 | 79.62 (75.93 - 83.49) | 75.88 (132040.27) | 71.79 (68.99) | 6.17 (4.5) |
|  |  | DEVICE USE ERROR | 273 | 12.86 (11.41 - 14.49) | 12.77 (2935.71) | 12.66 (11.45) | 3.66 (2) |
|  |  | FOREIGN BODY IN THROAT | 29 | 18.67 (12.94 - 26.93) | 18.65 (477.72) | 18.41 (13.54) | 4.2 (2.53) |
|  |  | CIRCUMSTANCE OR INFORMATION CAPABLE OF LEADING TO DEVICE USE ERROR | 6 | 7.44 (3.34 - 16.6) | 7.44 (33.27) | 7.41 (3.78) | 2.89 (1.22) |
|  |  | FOREIGN BODY IN RESPIRATORY TRACT | 5 | 5.13 (2.13 - 12.35) | 5.13 (16.56) | 5.11 (2.45) | 2.35 (0.69) |
|  |  | TRAUMATIC LUNG INJURY | 5 | 3.9 (1.62 - 9.39) | 3.9 (10.76) | 3.89 (1.87) | 1.96 (0.29) |
|  |  | FOREIGN BODY IN MOUTH | 5 | 107.67 (43.26 - 267.97) | 107.66 (488.31) | 99.58 (46.43) | 6.64 (4.93) |
|  |  | DEVICE MONITORING PROCEDURE NOT PERFORMED | 4 | 83.4 (30.36 - 229.14) | 83.39 (306.18) | 78.47 (33.69) | 6.29 (4.59) |
|  |  | STERNAL FRACTURE | 3 | 3.72 (1.2 - 11.57) | 3.72 (5.96) | 3.72 (1.44) | 1.89 (0.23) |
| INFECTIONS AND INFESTATIONS | 1273 | PNEUMONIA | 677 | 3.28 (3.04 - 3.54) | 3.24 (1053.57) | 3.24 (3.04) | 1.7 (0.03) |
|  |  | CANDIDA INFECTION | 296 | 29.18 (26 - 32.76) | 28.97 (7823.39) | 28.37 (25.75) | 4.83 (3.16) |
|  |  | ORAL CANDIDIASIS | 139 | 17.23 (14.57 - 20.38) | 17.18 (2090.67) | 16.97 (14.75) | 4.08 (2.42) |
|  |  | LARYNGITIS | 47 | 6.83 (5.12 - 9.09) | 6.82 (232.21) | 6.79 (5.34) | 2.76 (1.1) |
|  |  | CORONAVIRUS INFECTION | 38 | 8.57 (6.23 - 11.8) | 8.57 (252.36) | 8.52 (6.52) | 3.09 (1.42) |
|  |  | OROPHARYNGEAL CANDIDIASIS | 17 | 28.79 (17.8 - 46.55) | 28.77 (445.97) | 28.18 (18.85) | 4.82 (3.15) |
|  |  | OESOPHAGEAL CANDIDIASIS | 14 | 5.63 (3.33 - 9.52) | 5.63 (53.11) | 5.61 (3.62) | 2.49 (0.82) |
|  |  | ORAL FUNGAL INFECTION | 13 | 7.1 (4.11 - 12.24) | 7.09 (67.69) | 7.06 (4.47) | 2.82 (1.15) |
|  |  | MYCOBACTERIUM AVIUM COMPLEX INFECTION | 9 | 5.61 (2.92 - 10.8) | 5.61 (33.95) | 5.59 (3.23) | 2.48 (0.82) |
|  |  | SUSPECTED COVID-19 | 8 | 3.4 (1.7 - 6.8) | 3.4 (13.51) | 3.39 (1.9) | 1.76 (0.1) |
|  |  | ATYPICAL MYCOBACTERIAL INFECTION | 6 | 7.37 (3.3 - 16.45) | 7.37 (32.86) | 7.34 (3.75) | 2.88 (1.21) |
|  |  | FUNGAL OESOPHAGITIS | 3 | 6.46 (2.08 - 20.09) | 6.46 (13.78) | 6.43 (2.49) | 2.69 (1.02) |
|  |  | LOWER RESPIRATORY TRACT INFECTION BACTERIAL | 3 | 8.46 (2.72 - 26.32) | 8.46 (19.6) | 8.41 (3.25) | 3.07 (1.4) |
|  |  | PNEUMONIA PSEUDOMONAL | 3 | 3.4 (1.09 - 10.55) | 3.4 (5.06) | 3.39 (1.31) | 1.76 (0.09) |
| SURGICAL AND MEDICAL PROCEDURES | 787 | HOSPITALISATION | 592 | 6.36 (5.86 - 6.9) | 6.28 (2621.89) | 6.26 (5.84) | 2.65 (0.98) |
|  |  | STENT PLACEMENT | 28 | 5.19 (3.58 - 7.53) | 5.19 (94.35) | 5.17 (3.79) | 2.37 (0.7) |
|  |  | CATARACT OPERATION | 22 | 5.76 (3.79 - 8.76) | 5.76 (86.21) | 5.74 (4.04) | 2.52 (0.85) |
|  |  | CARDIAC PACEMAKER INSERTION | 21 | 5.23 (3.41 - 8.03) | 5.23 (71.48) | 5.21 (3.64) | 2.38 (0.71) |
|  |  | HERNIA REPAIR | 9 | 5.34 (2.77 - 10.28) | 5.34 (31.61) | 5.32 (3.08) | 2.41 (0.74) |
|  |  | EMERGENCY CARE | 8 | 4.55 (2.27 - 9.11) | 4.55 (22.07) | 4.54 (2.54) | 2.18 (0.51) |
|  |  | CHEMOTHERAPY | 8 | 3.95 (1.97 - 7.91) | 3.95 (17.56) | 3.94 (2.2) | 1.98 (0.31) |
|  |  | LUNG OPERATION | 8 | 14.84 (7.39 - 29.8) | 14.84 (102.11) | 14.69 (8.2) | 3.88 (2.21) |
|  |  | PALLIATIVE CARE | 7 | 7.88 (3.75 - 16.57) | 7.88 (41.79) | 7.84 (4.21) | 2.97 (1.3) |
|  |  | NEPHRECTOMY | 7 | 7.22 (3.43 - 15.17) | 7.22 (37.28) | 7.18 (3.86) | 2.84 (1.18) |
|  |  | LUNG LOBECTOMY | 6 | 26.63 (11.87 - 59.75) | 26.62 (145.03) | 26.11 (13.28) | 4.71 (3.03) |
|  |  | ENDOTRACHEAL INTUBATION | 6 | 3.33 (1.49 - 7.41) | 3.33 (9.73) | 3.32 (1.7) | 1.73 (0.06) |
|  |  | HEART VALVE REPLACEMENT | 6 | 6.34 (2.84 - 14.14) | 6.34 (26.86) | 6.31 (3.23) | 2.66 (0.99) |
|  |  | IMPLANTABLE DEFIBRILLATOR INSERTION | 5 | 5.53 (2.3 - 13.31) | 5.53 (18.47) | 5.51 (2.64) | 2.46 (0.79) |
|  |  | BRAIN OPERATION | 5 | 3.68 (1.53 - 8.85) | 3.68 (9.73) | 3.67 (1.76) | 1.88 (0.21) |
|  |  | SINUS OPERATION | 5 | 3.29 (1.37 - 7.92) | 3.29 (7.96) | 3.29 (1.58) | 1.72 (0.05) |
|  |  | QUARANTINE | 5 | 33.51 (13.79 - 81.41) | 33.51 (153.76) | 32.7 (15.56) | 5.03 (3.35) |
|  |  | PULMONARY RESECTION | 5 | 42.65 (17.5 - 103.93) | 42.64 (196.94) | 41.33 (19.62) | 5.37 (3.69) |
|  |  | ABDOMINAL OPERATION | 4 | 5.35 (2 - 14.29) | 5.35 (14.09) | 5.33 (2.34) | 2.41 (0.75) |
|  |  | BLADDER OPERATION | 4 | 5.29 (1.98 - 14.13) | 5.29 (13.86) | 5.27 (2.32) | 2.4 (0.73) |
|  |  | CANCER SURGERY | 4 | 6.5 (2.43 - 17.37) | 6.5 (18.53) | 6.47 (2.85) | 2.69 (1.03) |
|  |  | BLADDER CATHETERISATION | 4 | 6.54 (2.45 - 17.48) | 6.54 (18.69) | 6.52 (2.86) | 2.7 (1.03) |
|  |  | THERAPEUTIC PROCEDURE | 3 | 3.67 (1.18 - 11.41) | 3.67 (5.82) | 3.66 (1.42) | 1.87 (0.21) |
|  |  | WRIST SURGERY | 3 | 5.16 (1.66 - 16.03) | 5.16 (10.01) | 5.14 (1.99) | 2.36 (0.69) |
|  |  | SKIN NEOPLASM EXCISION | 3 | 6.08 (1.96 - 18.91) | 6.08 (12.68) | 6.06 (2.34) | 2.6 (0.93) |
|  |  | CARDIAC PACEMAKER REPLACEMENT | 3 | 10.37 (3.33 - 32.3) | 10.37 (25.2) | 10.3 (3.98) | 3.36 (1.69) |
|  |  | PANCREATECTOMY | 3 | 11.29 (3.62 - 35.18) | 11.29 (27.9) | 11.2 (4.33) | 3.49 (1.81) |
|  |  | SPLENECTOMY | 3 | 3.86 (1.24 - 11.98) | 3.86 (6.33) | 3.85 (1.49) | 1.94 (0.28) |
| GASTROINTESTINAL DISORDERS | 384 | ORAL PAIN | 67 | 4.42 (3.48 - 5.63) | 4.42 (176.72) | 4.41 (3.61) | 2.14 (0.47) |
|  |  | RETCHING | 57 | 4.14 (3.19 - 5.37) | 4.14 (135.14) | 4.13 (3.32) | 2.04 (0.38) |
|  |  | ORAL DISCOMFORT | 54 | 5.8 (4.44 - 7.58) | 5.8 (213.5) | 5.78 (4.62) | 2.53 (0.86) |
|  |  | ORAL MUCOSAL BLISTERING | 19 | 4.44 (2.83 - 6.97) | 4.44 (50.48) | 4.43 (3.04) | 2.15 (0.48) |
|  |  | TONGUE DISCOLOURATION | 18 | 5.87 (3.7 - 9.33) | 5.87 (72.45) | 5.85 (3.97) | 2.55 (0.88) |
|  |  | TONGUE DISCOMFORT | 18 | 6.75 (4.25 - 10.73) | 6.75 (87.74) | 6.72 (4.56) | 2.75 (1.08) |
|  |  | ORAL DISORDER | 17 | 3.48 (2.16 - 5.6) | 3.48 (29.92) | 3.47 (2.33) | 1.8 (0.13) |
|  |  | TONGUE COATED | 14 | 13.2 (7.8 - 22.36) | 13.2 (156.3) | 13.08 (8.42) | 3.71 (2.04) |
|  |  | COATING IN MOUTH | 13 | 39.08 (22.51 - 67.86) | 39.07 (468.34) | 37.97 (23.93) | 5.25 (3.58) |
|  |  | TONGUE BLISTERING | 11 | 7.17 (3.96 - 12.97) | 7.17 (58.05) | 7.13 (4.34) | 2.83 (1.17) |
|  |  | LIP PAIN | 10 | 5.21 (2.8 - 9.7) | 5.21 (33.91) | 5.2 (3.09) | 2.38 (0.71) |
|  |  | CHAPPED LIPS | 10 | 3.51 (1.89 - 6.54) | 3.51 (17.93) | 3.51 (2.09) | 1.81 (0.14) |
|  |  | GLOSSITIS | 9 | 3.79 (1.97 - 7.29) | 3.79 (18.42) | 3.78 (2.19) | 1.92 (0.25) |
|  |  | ORAL MUCOSAL ERUPTION | 8 | 8.08 (4.03 - 16.2) | 8.08 (49.35) | 8.04 (4.49) | 3.01 (1.34) |
|  |  | TONGUE ERYTHEMA | 7 | 14.02 (6.66 - 29.52) | 14.02 (83.71) | 13.88 (7.44) | 3.79 (2.12) |
|  |  | LIP DISORDER | 7 | 5.32 (2.53 - 11.19) | 5.32 (24.48) | 5.31 (2.85) | 2.41 (0.74) |
|  |  | TOOTH DISCOLOURATION | 7 | 3.48 (1.66 - 7.32) | 3.48 (12.36) | 3.48 (1.87) | 1.8 (0.13) |
|  |  | TEETH BRITTLE | 5 | 7.08 (2.94 - 17.04) | 7.08 (25.95) | 7.04 (3.38) | 2.82 (1.15) |
|  |  | TONGUE DRY | 5 | 5.54 (2.3 - 13.33) | 5.54 (18.51) | 5.52 (2.65) | 2.46 (0.8) |
|  |  | LIP DISCOLOURATION | 4 | 4.93 (1.85 - 13.17) | 4.93 (12.5) | 4.92 (2.16) | 2.3 (0.63) |
|  |  | OESOPHAGEAL DISCOMFORT | 4 | 8.56 (3.2 - 22.87) | 8.56 (26.52) | 8.51 (3.74) | 3.09 (1.42) |
|  |  | TONGUE HAEMORRHAGE | 4 | 5.58 (2.09 - 14.91) | 5.58 (14.99) | 5.56 (2.45) | 2.48 (0.81) |
|  |  | LIP EXFOLIATION | 4 | 4.65 (1.74 - 12.4) | 4.65 (11.4) | 4.63 (2.04) | 2.21 (0.54) |
|  |  | ANGINA BULLOSA HAEMORRHAGICA | 3 | 17.51 (5.61 - 54.72) | 17.51 (46.09) | 17.29 (6.67) | 4.11 (2.44) |
|  |  | GINGIVAL DISCOMFORT | 3 | 7.61 (2.45 - 23.67) | 7.61 (17.12) | 7.57 (2.93) | 2.92 (1.25) |
|  |  | SALIVA ALTERED | 3 | 5.01 (1.61 - 15.56) | 5.01 (9.58) | 4.99 (1.93) | 2.32 (0.65) |
|  |  | ORAL MUCOSAL DISCOLOURATION | 3 | 6.71 (2.16 - 20.88) | 6.71 (14.51) | 6.68 (2.59) | 2.74 (1.07) |
| RENAL AND URINARY DISORDERS | 332 | URINARY RETENTION | 175 | 8.11 (6.99 - 9.41) | 8.08 (1079.64) | 8.04 (7.1) | 3.01 (1.34) |
|  |  | DYSURIA | 104 | 4.23 (3.49 - 5.13) | 4.22 (255.13) | 4.21 (3.58) | 2.07 (0.41) |
|  |  | URINE FLOW DECREASED | 19 | 12 (7.64 - 18.85) | 11.99 (189.7) | 11.89 (8.15) | 3.57 (1.9) |
|  |  | MICTURITION DISORDER | 15 | 6.44 (3.88 - 10.7) | 6.44 (68.6) | 6.41 (4.2) | 2.68 (1.01) |
|  |  | URINARY TRACT DISORDER | 10 | 3.41 (1.83 - 6.34) | 3.41 (16.96) | 3.4 (2.02) | 1.77 (0.1) |
|  |  | BLADDER PAIN | 9 | 4.36 (2.26 - 8.39) | 4.36 (23.2) | 4.35 (2.51) | 2.12 (0.45) |
| GENERAL DISORDERS AND ADMINISTRATION SITE CONDITIONS | 295 | CHEST DISCOMFORT | 216 | 3.29 (2.88 - 3.76) | 3.27 (341.05) | 3.27 (2.92) | 1.71 (0.04) |
|  |  | SECRETION DISCHARGE | 74 | 9.86 (7.84 - 12.39) | 9.84 (583.59) | 9.78 (8.07) | 3.29 (1.62) |
|  |  | MUCOSAL DISCOLOURATION | 5 | 11.92 (4.94 - 28.75) | 11.92 (49.56) | 11.82 (5.66) | 3.56 (1.89) |
| NEOPLASMS BENIGN, MALIGNANT AND UNSPECIFIED (INCL CYSTS AND POLYPS) | 229 | LUNG NEOPLASM MALIGNANT | 192 | 6.76 (5.86 - 7.79) | 6.73 (932.7) | 6.7 (5.95) | 2.74 (1.08) |
|  |  | BRAIN NEOPLASM MALIGNANT | 11 | 3.5 (1.94 - 6.33) | 3.5 (19.62) | 3.5 (2.13) | 1.81 (0.14) |
|  |  | LUNG CARCINOMA CELL TYPE UNSPECIFIED STAGE IV | 10 | 4.16 (2.23 - 7.73) | 4.16 (23.9) | 4.15 (2.47) | 2.05 (0.39) |
|  |  | SMALL CELL LUNG CANCER | 7 | 8.12 (3.86 - 17.08) | 8.12 (43.45) | 8.08 (4.34) | 3.01 (1.35) |
|  |  | LUNG CARCINOMA CELL TYPE UNSPECIFIED STAGE III | 5 | 8.07 (3.35 - 19.44) | 8.07 (30.77) | 8.02 (3.85) | 3 (1.34) |
|  |  | LUNG CARCINOMA CELL TYPE UNSPECIFIED RECURRENT | 4 | 10.3 (3.85 - 27.56) | 10.3 (33.33) | 10.23 (4.49) | 3.35 (1.68) |
| INVESTIGATIONS | 200 | OXYGEN SATURATION DECREASED | 136 | 3.89 (3.29 - 4.61) | 3.88 (290.37) | 3.87 (3.36) | 1.95 (0.29) |
|  |  | OXYGEN SATURATION ABNORMAL | 11 | 6.58 (3.64 - 11.9) | 6.58 (51.75) | 6.55 (3.99) | 2.71 (1.04) |
|  |  | CORONAVIRUS TEST POSITIVE | 11 | 15.39 (8.49 - 27.89) | 15.39 (146.25) | 15.22 (9.25) | 3.93 (2.26) |
|  |  | TOTAL LUNG CAPACITY DECREASED | 10 | 9.97 (5.35 - 18.57) | 9.97 (80.05) | 9.9 (5.88) | 3.31 (1.64) |
|  |  | OXYGEN CONSUMPTION INCREASED | 5 | 3.32 (1.38 - 7.99) | 3.32 (8.1) | 3.32 (1.59) | 1.73 (0.06) |
|  |  | BIOPSY LUNG | 4 | 15.97 (5.96 - 42.81) | 15.97 (55.45) | 15.79 (6.92) | 3.98 (2.31) |
|  |  | CARBON DIOXIDE INCREASED | 4 | 4.57 (1.71 - 12.21) | 4.57 (11.13) | 4.56 (2.01) | 2.19 (0.52) |
|  |  | EMERGENCY CARE EXAMINATION | 4 | 9.73 (3.64 - 26.02) | 9.73 (31.1) | 9.66 (4.24) | 3.27 (1.6) |
|  |  | INSPIRATORY CAPACITY DECREASED | 3 | 22.14 (7.07 - 69.3) | 22.14 (59.54) | 21.79 (8.38) | 4.45 (2.77) |
|  |  | SPUTUM ABNORMAL | 3 | 6.87 (2.21 - 21.35) | 6.86 (14.95) | 6.83 (2.64) | 2.77 (1.1) |
|  |  | PO2 DECREASED | 3 | 3.78 (1.22 - 11.73) | 3.78 (6.11) | 3.77 (1.46) | 1.91 (0.25) |
|  |  | SPIROMETRY ABNORMAL | 3 | 8.76 (2.81 - 27.25) | 8.76 (20.47) | 8.7 (3.37) | 3.12 (1.45) |
|  |  | OXYGEN CONSUMPTION DECREASED | 3 | 7.11 (2.29 - 22.12) | 7.11 (15.67) | 7.08 (2.74) | 2.82 (1.15) |
| NERVOUS SYSTEM DISORDERS | 71 | TASTE DISORDER | 67 | 6.1 (4.8 - 7.76) | 6.09 (284.03) | 6.07 (4.96) | 2.6 (0.94) |
|  |  | VOCAL CORD PARALYSIS | 4 | 3.37 (1.26 - 8.99) | 3.37 (6.64) | 3.36 (1.48) | 1.75 (0.08) |
| EYE DISORDERS | 48 | GLAUCOMA | 45 | 3.53 (2.64 - 4.74) | 3.53 (81.45) | 3.52 (2.76) | 1.82 (0.15) |
|  |  | HALO VISION | 3 | 3.94 (1.27 - 12.24) | 3.94 (6.56) | 3.93 (1.52) | 1.98 (0.31) |
| IMMUNE SYSTEM DISORDERS | 23 | MULTIPLE ALLERGIES | 23 | 4.59 (3.05 - 6.91) | 4.59 (64.31) | 4.58 (3.25) | 2.19 (0.53) |
| PSYCHIATRIC DISORDERS | 18 | NEAR DEATH EXPERIENCE | 18 | 9.76 (6.14 - 15.51) | 9.75 (140.37) | 9.69 (6.57) | 3.28 (1.61) |
| PRODUCT ISSUES | 10 | PRODUCT AFTER TASTE | 10 | 24.15 (12.92 - 45.14) | 24.14 (217.85) | 23.73 (14.06) | 4.57 (2.9) |
| SOCIAL CIRCUMSTANCES | 6 | DEPENDENCE ON OXYGEN THERAPY | 3 | 11.07 (3.55 - 34.49) | 11.07 (27.25) | 10.98 (4.24) | 3.46 (1.78) |
|  |  | CARDIAC ASSISTANCE DEVICE USER | 3 | 11.83 (3.8 - 36.88) | 11.83 (29.49) | 11.74 (4.53) | 3.55 (1.88) |

Note: N, counts, ROR, reporting odds ratio; PRR, proportional reporting ratio; IC, information component; EBGM, Empirical Bayes Geometric Mean; PT, Preferred Term.
