# Supplementary material for: Adverse event mining for Breztri and Trelegy Ellipta based on the three international pharmacovigilance databases
Source: Medicine (Baltimore). 2026 Jun 5;105(23):e49162. doi: 10.1097/MD.0000000000049162 (PMC13246110; doi:10.1097/MD.0000000000049162)
Supplement: Supplementary file 3 [file medi-105-e49162-s003.docx]

Table S3 Signal-positive ADE PTs for Breztri of JADER

| soc_name_en | N | pt_name_en | N | ROR (95%Cl) | PRR (Chi-Square Value) | EBGM (EBGM05) | IC (IC025) |
| --- | --- | --- | --- | --- | --- | --- | --- |
| Infections and infestations | 31 | Pneumonia | 28 | 15.03 (10.04 - 22.52) | 12.8 (308.02) | 12.78 (8.53) | 3.68 (2) |
|  |  | Pneumonia bacterial | 3 | 10.73 (3.42 - 33.6) | 10.56 (25.98) | 10.55 (3.37) | 3.4 (1.72) |
| Respiratory, thoracic and mediastinal disorders | 22 | Chronic obstructive pulmonary disease | 13 | 516.8 (289.76 - 921.71) | 478.7 (5885.82) | 454.63 (254.91) | 8.83 (7.14) |
|  |  | Dyspnoea | 6 | 6.65 (2.95 - 15.02) | 6.46 (27.82) | 6.46 (2.86) | 2.69 (1.01) |
|  |  | Emphysema | 3 | 191.19 (60.36 - 605.64) | 187.95 (546.53) | 184.14 (58.13) | 7.52 (5.83) |
| Cardiac disorders | 11 | Cardiac failure | 8 | 7.82 (3.84 - 15.89) | 7.51 (45.35) | 7.5 (3.69) | 2.91 (1.23) |
|  |  | Arrhythmia | 3 | 11.51 (3.67 - 36.06) | 11.33 (28.26) | 11.32 (3.61) | 3.5 (1.82) |
| General disorders and administration site conditions | 8 | Death | 10 | 8.7 (4.59 - 16.47) | 8.26 (64.19) | 8.25 (4.36) | 3.04 (1.36) |
| Nervous system disorders | 8 | Tremor | 3 | 14.65 (4.68 - 45.92) | 14.42 (37.45) | 14.4 (4.59) | 3.85 (2.17) |
| Neoplasms benign, malignant and unspecified (incl cysts and polyps) | 3 | Lung neoplasm malignant | 3 | 20.24 (6.46 - 63.46) | 19.92 (53.82) | 19.87 (6.34) | 4.31 (2.63) |
| Renal and urinary disorders | 3 | Urinary retention | 8 | 15.34 (7.54 - 31.19) | 14.68 (102.17) | 14.66 (7.21) | 3.87 (2.19) |

Note: N, counts, ROR, reporting odds ratio; PRR, proportional reporting ratio; IC, information component; EBGM, Empirical Bayes Geometric Mean.
