# Supplementary material for: Adverse event mining for Breztri and Trelegy Ellipta based on the three international pharmacovigilance databases
Source: Medicine (Baltimore). 2026 Jun 5;105(23):e49162. doi: 10.1097/MD.0000000000049162 (PMC13246110; doi:10.1097/MD.0000000000049162)
Supplement: Supplementary file 4 [file medi-105-e49162-s004.docx]

Table S4 Signal-positive ADE PTs for Trelegy Ellipta of JADER

| soc_name_en | N | pt_name_en | N | ROR (95%Cl) | PRR (Chi-Square Value) | EBGM (EBGM05) | IC (IC025) |
| --- | --- | --- | --- | --- | --- | --- | --- |
| Infections and infestations | 167 | Pneumonia | 142 | 29.47 (24.3 - 35.75) | 21.8 (2833.45) | 21.65 (17.85) | 4.44 (2.77) |
|  |  | Oesophageal candidiasis | 9 | 72.57 (37.26 - 141.33) | 71.34 (609.92) | 69.72 (35.8) | 6.12 (4.45) |
|  |  | Pneumonia bacterial | 7 | 8.34 (3.95 - 17.59) | 8.24 (44.48) | 8.22 (3.9) | 3.04 (1.37) |
|  |  | Atypical mycobacterial infection | 3 | 10.74 (3.44 - 33.47) | 10.68 (26.25) | 10.65 (3.42) | 3.41 (1.74) |
|  |  | Bronchopulmonary aspergillosis | 3 | 7 (2.25 - 21.79) | 6.96 (15.29) | 6.95 (2.23) | 2.8 (1.12) |
|  |  | Gastrointestinal fungal infection | 3 | 413.11 (123.27 - 1384.43) | 410.76 (1079.16) | 361.59 (107.9) | 8.5 (6.73) |
| Respiratory, thoracic and mediastinal disorders | 50 | Dyspnoea | 12 | 4.39 (2.48 - 7.79) | 4.32 (30.71) | 4.31 (2.43) | 2.11 (0.44) |
|  |  | Chronic obstructive pulmonary disease | 11 | 136.99 (74.42 - 252.14) | 134.15 (1391.97) | 128.47 (69.8) | 7.01 (5.33) |
|  |  | Asthma | 7 | 10.34 (4.9 - 21.82) | 10.22 (58.08) | 10.18 (4.83) | 3.35 (1.68) |
|  |  | Cough | 5 | 6.82 (2.83 - 16.48) | 6.77 (24.56) | 6.76 (2.8) | 2.76 (1.08) |
|  |  | Respiratory failure | 4 | 3.93 (1.47 - 10.53) | 3.91 (8.68) | 3.91 (1.46) | 1.97 (0.3) |
|  |  | Pneumothorax | 4 | 8.06 (3.01 - 21.58) | 8.01 (24.48) | 7.99 (2.98) | 3 (1.33) |
|  |  | Productive cough | 4 | 51.88 (19.24 - 139.9) | 51.49 (194.74) | 50.64 (18.78) | 5.66 (3.98) |
|  |  | Organising pneumonia | 3 | 6.24 (2 - 19.44) | 6.21 (13.1) | 6.2 (1.99) | 2.63 (0.96) |
| Renal and urinary disorders | 43 | Urinary retention | 43 | 28.81 (21.07 - 39.41) | 26.54 (1050.97) | 26.32 (19.24) | 4.72 (3.05) |
| Surgical and medical procedures | 20 | Hospitalisation | 20 | 20.64 (13.18 - 32.32) | 19.9 (357.26) | 19.77 (12.63) | 4.31 (2.63) |
| Cardiac disorders | 17 | Arrhythmia | 9 | 11.56 (5.97 - 22.36) | 11.38 (84.99) | 11.34 (5.86) | 3.5 (1.83) |
|  |  | Atrial fibrillation | 8 | 9.02 (4.48 - 18.16) | 8.9 (56.05) | 8.88 (4.41) | 3.15 (1.48) |
| Injury, poisoning and procedural complications | 13 | Foreign body in throat | 13 | 436.38 (242.59 - 784.99) | 425.64 (4825.86) | 373.07 (207.39) | 8.54 (6.85) |
| Eye disorders | 11 | Glaucoma | 7 | 25.01 (11.83 - 52.88) | 24.69 (157.91) | 24.5 (11.59) | 4.61 (2.94) |
|  |  | Cataract | 4 | 7.9 (2.95 - 21.15) | 7.84 (23.85) | 7.83 (2.92) | 2.97 (1.3) |
| Metabolism and nutrition disorders | 9 | Hypokalaemia | 5 | 3.52 (1.46 - 8.5) | 3.5 (8.93) | 3.49 (1.45) | 1.8 (0.13) |
|  |  | Diabetes mellitus | 4 | 3.73 (1.39 - 9.97) | 3.71 (7.91) | 3.7 (1.38) | 1.89 (0.22) |
| Skin and subcutaneous tissue disorders | 3 | Angioedema | 3 | 8.04 (2.58 - 25.06) | 8 (18.35) | 7.99 (2.56) | 3 (1.32) |
| Neoplasms benign, malignant and unspecified (incl cysts and polyps) | 3 | Neoplasm malignant | 3 | 8.83 (2.83 - 27.5) | 8.78 (20.64) | 8.76 (2.81) | 3.13 (1.46) |
| Gastrointestinal disorders | 3 | Haematemesis | 3 | 7.68 (2.47 - 23.93) | 7.65 (17.3) | 7.63 (2.45) | 2.93 (1.26) |

Note: N, counts, ROR, reporting odds ratio; PRR, proportional reporting ratio; IC, information component; EBGM, Empirical Bayes Geometric Mean.
