# Supplementary material for: Adverse event mining for Breztri and Trelegy Ellipta based on the three international pharmacovigilance databases
Source: Medicine (Baltimore). 2026 Jun 5;105(23):e49162. doi: 10.1097/MD.0000000000049162 (PMC13246110; doi:10.1097/MD.0000000000049162)
Supplement: Supplementary file 5 [file medi-105-e49162-s005.docx]

Table S5 Signal-positive ADE PTs for Breztri of CAVR database

| soc_name_en | N | pt_name_en | n | ROR (95%Cl) | PRR (Chi-Square Value) | EBGM (EBGM05) | IC (IC025) |
| --- | --- | --- | --- | --- | --- | --- | --- |
| Respiratory, thoracic and mediastinal disorders | 13 | Dyspnoea | 7 | 6.67 (3.09 - 14.38) | 6.28 (31.4) | 6.28 (2.91) | 2.65 (0.96) |
|  |  | Asthma | 6 | 16.98 (7.44 - 38.76) | 16.03 (84.85) | 16.03 (7.02) | 4 (2.31) |
| Product issues | 6 | Device delivery system issue | 3 | 503.02 (158.42 - 1597.2) | 488.11 (1441.32) | 482.4 (151.92) | 8.91 (7.21) |
|  |  | Device defective | 3 | 201.67 (63.76 - 637.82) | 195.71 (578.47) | 194.78 (61.59) | 7.61 (5.91) |
| Injury, poisoning and procedural complications | 5 | Device use issue | 5 | 565.32 (228.78 - 1396.92) | 537.38 (2642.67) | 530.47 (214.68) | 9.05 (7.35) |
| Social circumstances | 3 | Loss of personal independence in daily activities | 3 | 15.43 (4.89 - 48.68) | 15 (39.27) | 15 (4.75) | 3.91 (2.22) |
| Vascular disorders | 3 | Hypertension | 3 | 7.82 (2.48 - 24.67) | 7.62 (17.31) | 7.62 (2.41) | 2.93 (1.24) |

Note: N, counts, ROR, reporting odds ratio; PRR, proportional reporting ratio; IC, information component; EBGM, Empirical Bayes Geometric Mean.
