# Supplementary material for: Adverse event mining for Breztri and Trelegy Ellipta based on the three international pharmacovigilance databases
Source: Medicine (Baltimore). 2026 Jun 5;105(23):e49162. doi: 10.1097/MD.0000000000049162 (PMC13246110; doi:10.1097/MD.0000000000049162)
Supplement: Supplementary file 6 [file medi-105-e49162-s006.docx]

Table S6 Signal-positive ADE PTs for Trelegy Ellipta of CAVR database

| soc_name_en | N | pt_name_en | N | ROR (95%Cl) | PRR (Chi-Square Value) | EBGM (EBGM05) | IC (IC025) |
| --- | --- | --- | --- | --- | --- | --- | --- |
| Respiratory, thoracic and mediastinal disorders | 580 | Dyspnoea | 115 | 3.66 (3.03 - 4.41) | 3.55 (212.74) | 3.55 (2.94) | 1.83 (0.16) |
|  |  | Wheezing | 79 | 12.9 (10.31 - 16.15) | 12.58 (836.53) | 12.48 (9.97) | 3.64 (1.97) |
|  |  | Productive cough | 49 | 9.11 (6.86 - 12.09) | 8.98 (345.7) | 8.92 (6.72) | 3.16 (1.49) |
|  |  | Lung disorder | 35 | 12.41 (8.88 - 17.34) | 12.27 (359.66) | 12.18 (8.71) | 3.61 (1.94) |
|  |  | Respiratory symptom | 34 | 145.24 (101.87 - 207.07) | 143.57 (4370.51) | 130.43 (91.49) | 7.03 (5.35) |
|  |  | Haemoptysis | 31 | 26.75 (18.72 - 38.24) | 26.48 (746.42) | 26.01 (18.2) | 4.7 (3.03) |
|  |  | Pulmonary alveolar haemorrhage | 30 | 337.6 (226.4 - 503.4) | 334.16 (8061.01) | 270.5 (181.4) | 8.08 (6.4) |
|  |  | Pulmonary congestion | 30 | 28.82 (20.04 - 41.45) | 28.54 (781.75) | 27.99 (19.47) | 4.81 (3.14) |
|  |  | Pulmonary fibrosis | 29 | 23.56 (16.29 - 34.06) | 23.34 (610.17) | 22.97 (15.89) | 4.52 (2.85) |
|  |  | Pulmonary vasculitis | 29 | 1062.36 (656.08 - 1720.24) | 1051.88 (17462.31) | 603.71 (372.83) | 9.24 (7.54) |
|  |  | Dyspnoea exertional | 29 | 10.29 (7.13 - 14.86) | 10.2 (239.22) | 10.14 (7.02) | 3.34 (1.67) |
|  |  | Pulmonary embolism | 28 | 7.65 (5.27 - 11.12) | 7.59 (159.58) | 7.56 (5.2) | 2.92 (1.25) |
|  |  | Increased bronchial secretion | 28 | 72.44 (49.47 - 106.07) | 71.76 (1859.48) | 68.34 (46.67) | 6.09 (4.42) |
|  |  | Hypoxia | 17 | 12.42 (7.69 - 20.04) | 12.35 (175.9) | 12.25 (7.59) | 3.62 (1.95) |
|  |  | Sinus disorder | 8 | 9.53 (4.75 - 19.11) | 9.5 (60.47) | 9.45 (4.71) | 3.24 (1.57) |
|  |  | Choking | 5 | 5.07 (2.1 - 12.2) | 5.06 (16.23) | 5.04 (2.09) | 2.33 (0.67) |
|  |  | Lung opacity | 4 | 16.81 (6.27 - 45.09) | 16.79 (58.71) | 16.61 (6.19) | 4.05 (2.38) |
| General disorders and administration site conditions | 258 | Death | 128 | 4.15 (3.48 - 4.96) | 4.01 (292.15) | 4.01 (3.36) | 2 (0.34) |
|  |  | Therapeutic product effect incomplete | 61 | 3.86 (2.99 - 4.97) | 3.8 (126.01) | 3.79 (2.94) | 1.92 (0.25) |
|  |  | Chest discomfort | 30 | 3.81 (2.66 - 5.46) | 3.78 (61.35) | 3.77 (2.63) | 1.92 (0.25) |
|  |  | Nodule | 17 | 17.94 (11.1 - 28.98) | 17.84 (266.95) | 17.63 (10.91) | 4.14 (2.47) |
|  |  | Multiple organ dysfunction syndrome | 15 | 26.26 (15.74 - 43.82) | 26.13 (356.05) | 25.68 (15.39) | 4.68 (3.01) |
|  |  | Ulcer | 7 | 8.55 (4.06 - 17.98) | 8.53 (46.26) | 8.48 (4.03) | 3.08 (1.42) |
| Investigations | 188 | Full blood count abnormal | 47 | 23.95 (17.91 - 32.03) | 23.59 (1000.51) | 23.21 (17.36) | 4.54 (2.87) |
|  |  | Forced expiratory volume decreased | 18 | 18.94 (11.88 - 30.2) | 18.83 (300.06) | 18.6 (11.67) | 4.22 (2.55) |
|  |  | Total lung capacity abnormal | 18 | 656.91 (375.35 - 1149.67) | 652.89 (8016.38) | 447.03 (255.43) | 8.8 (7.09) |
|  |  | Spirometry abnormal | 17 | 234.83 (140.4 - 392.78) | 233.48 (3377.8) | 200.54 (119.9) | 7.65 (5.96) |
|  |  | Antineutrophil cytoplasmic antibody positive | 16 | 291.76 (170.17 - 500.23) | 290.17 (3826.11) | 240.95 (140.54) | 7.91 (6.22) |
|  |  | Blood phosphorus increased | 12 | 104.57 (58.12 - 188.14) | 104.14 (1141.81) | 97.07 (53.95) | 6.6 (4.92) |
|  |  | Blood uric acid increased | 11 | 57.84 (31.62 - 105.82) | 57.63 (588.22) | 55.41 (30.29) | 5.79 (4.12) |
|  |  | Blood cholesterol increased | 11 | 6.86 (3.79 - 12.42) | 6.84 (54.61) | 6.81 (3.76) | 2.77 (1.1) |
|  |  | Activated partial thromboplastin time prolonged | 6 | 44.07 (19.54 - 99.38) | 43.98 (244.41) | 42.68 (18.92) | 5.42 (3.74) |
|  |  | Troponin increased | 6 | 11.89 (5.32 - 26.58) | 11.87 (59.24) | 11.78 (5.27) | 3.56 (1.89) |
|  |  | Blood calcium increased | 5 | 17.84 (7.38 - 43.14) | 17.82 (78.38) | 17.61 (7.28) | 4.14 (2.47) |
|  |  | Blood test abnormal | 5 | 4.18 (1.74 - 10.07) | 4.18 (12.06) | 4.17 (1.73) | 2.06 (0.39) |
|  |  | Total lung capacity decreased | 5 | 27.35 (11.28 - 66.32) | 27.31 (124.34) | 26.81 (11.06) | 4.74 (3.07) |
|  |  | Liver function test increased | 4 | 3.44 (1.29 - 9.19) | 3.44 (6.91) | 3.43 (1.29) | 1.78 (0.11) |
|  |  | Transaminases increased | 4 | 14.38 (5.37 - 38.53) | 14.36 (49.23) | 14.23 (5.31) | 3.83 (2.16) |
|  |  | Blood sodium decreased | 3 | 5.29 (1.7 - 16.44) | 5.28 (10.39) | 5.27 (1.69) | 2.4 (0.73) |
| Psychiatric disorders | 141 | Sleep disorder due to a general medical condition | 67 | 48.06 (37.58 - 61.48) | 46.99 (2920.24) | 45.51 (35.58) | 5.51 (3.84) |
|  |  | Anxiety | 41 | 3.68 (2.7 - 5.01) | 3.64 (78.68) | 3.64 (2.67) | 1.86 (0.2) |
|  |  | Sleep disorder | 25 | 6.84 (4.61 - 10.15) | 6.79 (122.98) | 6.76 (4.56) | 2.76 (1.09) |
|  |  | Mental disorder | 8 | 5.87 (2.93 - 11.77) | 5.86 (32.13) | 5.84 (2.91) | 2.55 (0.88) |
| Nervous system disorders | 93 | Neuritis | 29 | 567.56 (368.55 - 874.02) | 561.96 (11622.33) | 402.47 (261.35) | 8.65 (6.96) |
|  |  | Neurological symptom | 29 | 122.94 (83.98 - 179.95) | 121.73 (3197.58) | 112.17 (76.63) | 6.81 (5.14) |
|  |  | Myasthenia gravis | 14 | 64.6 (37.77 - 110.51) | 64.3 (834.55) | 61.55 (35.98) | 5.94 (4.27) |
|  |  | Neuralgia | 12 | 9.53 (5.4 - 16.84) | 9.5 (90.69) | 9.44 (5.35) | 3.24 (1.57) |
|  |  | Mental impairment | 9 | 13.19 (6.84 - 25.45) | 13.15 (100.15) | 13.04 (6.76) | 3.7 (2.04) |
| Vascular disorders | 85 | Vasculitis | 32 | 47.12 (33.07 - 67.14) | 46.62 (1383.26) | 45.16 (31.7) | 5.5 (3.83) |
|  |  | Thrombosis | 28 | 9.74 (6.7 - 14.15) | 9.66 (216.01) | 9.6 (6.61) | 3.26 (1.6) |
|  |  | Arteriosclerosis | 17 | 65.9 (40.47 - 107.32) | 65.53 (1032.48) | 62.67 (38.49) | 5.97 (4.3) |
|  |  | Aortic stenosis | 5 | 37.49 (15.41 - 91.17) | 37.42 (172.69) | 36.48 (15) | 5.19 (3.51) |
|  |  | Superficial vein thrombosis | 3 | 26.38 (8.42 - 82.72) | 26.36 (71.85) | 25.9 (8.26) | 4.69 (3.01) |
| Social circumstances | 82 | Loss of personal independence in daily activities | 82 | 14.61 (11.72 - 18.21) | 14.23 (1000.36) | 14.1 (11.31) | 3.82 (2.15) |
| Surgical and medical procedures | 76 | Hospitalisation | 29 | 3.74 (2.59 - 5.39) | 3.71 (57.43) | 3.7 (2.57) | 1.89 (0.22) |
|  |  | Sleep disorder therapy | 10 | 1290.39 (547.6 - 3040.73) | 1286 (6725.75) | 674.1 (286.07) | 9.4 (7.62) |
|  |  | Analgesic therapy | 10 | 946.29 (424.77 - 2108.08) | 943.07 (5646.44) | 566.24 (254.18) | 9.15 (7.39) |
|  |  | Drug therapy | 9 | 982.35 (419.58 - 2299.92) | 979.34 (5197.7) | 579.11 (247.35) | 9.18 (7.41) |
|  |  | Breast conserving surgery | 7 | 152.7 (69.96 - 333.29) | 152.34 (950.13) | 137.63 (63.06) | 7.1 (5.4) |
|  |  | Anticoagulant therapy | 6 | 447.63 (178.64 - 1121.62) | 446.72 (2027.93) | 339.74 (135.59) | 8.41 (6.65) |
|  |  | Antacid therapy | 5 | 1417.01 (410 - 4897.33) | 1414.6 (3531.51) | 707.8 (204.8) | 9.47 (7.59) |
| Metabolism and nutrition disorders | 73 | Hyponatraemia | 18 | 14.31 (8.98 - 22.79) | 14.23 (219.21) | 14.09 (8.85) | 3.82 (2.15) |
|  |  | Hypophosphataemia | 13 | 88.38 (50.41 - 154.94) | 87.99 (1052.6) | 82.9 (47.28) | 6.37 (4.7) |
|  |  | Diabetes mellitus | 11 | 3.47 (1.92 - 6.27) | 3.46 (19.18) | 3.45 (1.91) | 1.79 (0.12) |
|  |  | Hyperphosphataemia | 10 | 189.25 (97.74 - 366.47) | 188.61 (1646.67) | 166.54 (86.01) | 7.38 (5.68) |
|  |  | Iron deficiency | 9 | 33 (17.02 - 63.96) | 32.9 (272.05) | 32.17 (16.6) | 5.01 (3.33) |
|  |  | Hyperlipidaemia | 6 | 37.97 (16.86 - 85.48) | 37.89 (209.89) | 36.93 (16.4) | 5.21 (3.53) |
|  |  | Gout | 6 | 6.22 (2.79 - 13.89) | 6.21 (26.14) | 6.19 (2.77) | 2.63 (0.96) |
| Immune system disorders | 73 | Eosinophilic granulomatosis with polyangiitis | 22 | 141.24 (91 - 219.21) | 140.19 (2766.28) | 127.64 (82.24) | 7 (5.32) |
|  |  | Dust allergy | 17 | 94.11 (57.53 - 153.96) | 93.57 (1460.42) | 87.83 (53.69) | 6.46 (4.78) |
|  |  | Mite allergy | 17 | 196.65 (118.27 - 326.97) | 195.51 (2890.44) | 171.89 (103.38) | 7.43 (5.74) |
|  |  | Mycotic allergy | 17 | 262.91 (156.51 - 441.65) | 261.39 (3722.09) | 220.78 (131.43) | 7.79 (6.1) |
| Infections and infestations | 47 | Sepsis | 16 | 4.54 (2.78 - 7.43) | 4.52 (43.85) | 4.51 (2.76) | 2.17 (0.51) |
|  |  | Appendicitis | 14 | 21.93 (12.92 - 37.23) | 21.84 (274.16) | 21.52 (12.68) | 4.43 (2.76) |
|  |  | Bacterial infection | 11 | 8.92 (4.92 - 16.15) | 8.89 (76.54) | 8.84 (4.88) | 3.14 (1.48) |
|  |  | Oral candidiasis | 3 | 4.23 (1.36 - 13.13) | 4.22 (7.36) | 4.21 (1.36) | 2.07 (0.41) |
|  |  | Bacteroides infection | 3 | 1062.03 (237.59 - 4747.4) | 1060.95 (1815.35) | 606.69 (135.72) | 9.24 (7.29) |
| Cardiac disorders | 45 | Arteriosclerosis coronary artery | 17 | 67.18 (41.25 - 109.43) | 66.8 (1052.27) | 63.83 (39.19) | 6 (4.32) |
|  |  | Ventricular fibrillation | 14 | 63.77 (37.29 - 109.08) | 63.48 (823.97) | 60.79 (35.54) | 5.93 (4.25) |
|  |  | Cardiogenic shock | 14 | 60.48 (35.38 - 103.38) | 60.2 (781.77) | 57.78 (33.8) | 5.85 (4.18) |
| Gastrointestinal disorders | 42 | Appendicolith | 15 | 646.3 (350.67 - 1191.15) | 643 (6610.4) | 442.37 (240.02) | 8.79 (7.07) |
|  |  | Ascites | 14 | 15.93 (9.39 - 27.01) | 15.86 (192.77) | 15.69 (9.25) | 3.97 (2.3) |
|  |  | Dry mouth | 13 | 3.72 (2.16 - 6.43) | 3.71 (25.73) | 3.71 (2.15) | 1.89 (0.22) |
| Injury, poisoning and procedural complications | 25 | Intentional product misuse | 13 | 8.22 (4.76 - 14.2) | 8.19 (81.65) | 8.15 (4.72) | 3.03 (1.36) |
|  |  | Incorrect route of product administration | 12 | 15.94 (9.01 - 28.19) | 15.88 (165.5) | 15.71 (8.89) | 3.97 (2.3) |
| Blood and lymphatic system disorders | 25 | Normochromic normocytic anaemia | 17 | 409.96 (238.73 - 704.02) | 407.6 (5352.94) | 316.65 (184.39) | 8.31 (6.61) |
|  |  | Eosinophilia | 8 | 16.69 (8.3 - 33.53) | 16.64 (116.27) | 16.46 (8.19) | 4.04 (2.37) |
| Endocrine disorders | 21 | Hypothyroidism | 21 | 19.57 (12.71 - 30.16) | 19.44 (362.51) | 19.19 (12.46) | 4.26 (2.59) |
| Eye disorders | 17 | Conjunctivitis allergic | 17 | 278.02 (165.12 - 468.11) | 276.42 (3902.66) | 231.4 (137.43) | 7.85 (6.16) |
| Product issues | 15 | Product complaint | 15 | 8.89 (5.35 - 14.79) | 8.85 (103.89) | 8.8 (5.29) | 3.14 (1.47) |
| Neoplasms benign, malignant and unspecified (incl cysts and polyps) | 14 | Breast cancer | 8 | 3.78 (1.89 - 7.57) | 3.77 (16.27) | 3.76 (1.88) | 1.91 (0.25) |
|  |  | Lung neoplasm malignant | 6 | 3.41 (1.53 - 7.6) | 3.4 (10.16) | 3.4 (1.52) | 1.76 (0.1) |
| Renal and urinary disorders | 11 | End stage renal disease | 6 | 32.71 (14.55 - 73.54) | 32.64 (179.91) | 31.93 (14.2) | 5 (3.32) |
|  |  | Urinary retention | 5 | 4.61 (1.92 - 11.11) | 4.61 (14.08) | 4.6 (1.91) | 2.2 (0.53) |
| Skin and subcutaneous tissue disorders | 6 | Capillaritis | 6 | 1214.99 (408.07 - 3617.5) | 1212.51 (3910.91) | 653.35 (219.44) | 9.35 (7.52) |

Note: N, counts, ROR, reporting odds ratio; PRR, proportional reporting ratio; IC, information component; EBGM, Empirical Bayes Geometric Mean.
